# Trends in socioeconomic inequalities in mortality in small areas of 33 Spanish cities

**DOI:** 10.1186/s12889-016-3190-y

**Published:** 2016-07-29

**Authors:** Marc Marí-Dell’Olmo, Mercè Gotsens, Laia Palència, Maica Rodríguez-Sanz, Miguel A. Martinez-Beneito, Mónica Ballesta, Montse Calvo, Lluís Cirera, Antonio Daponte, Felicitas Domínguez-Berjón, Ana Gandarillas, Natividad Izco Goñi, Carmen Martos, Conchi Moreno-Iribas, Andreu Nolasco, Diego Salmerón, Margarita Taracido, Carme Borrell

**Affiliations:** 1 CIBER Epidemiología y Salud Pública (CIBERESP), Madrid, Spain; 2 Agència de Salut Pública de Barcelona, Plaça Lesseps 1, 08023 Barcelona, Spain; 3 Institut d’Investigació Biomèdica (IIB Sant Pau), Barcelona, Spain; 4 Universitat Rovira i Virgili, Tarragona, Spain; 5 Fundación para el fomento de la investigación sanitaria y biomédica de la Comunidad Valenciana (FISABIO), Valencia, Spain; 6 Department of Epidemiology, Murcia Regional Health Council, IMIB-Arrixaca, Murcia, Spain; 7 Estudios e investigación Sanitaria, Departamento de Sanidad y Consumo, Gobierno Vasco, Vitoria-Gasteiz, Spain; 8 Observatorio de Salud y Medio Ambiente de Andalucía (OSMAN), Escuela Andaluza de Salud Pública (EASP), Granada, Spain; 9 Subdirección de Promoción de la Salud y Prevención, Consejería de Sanidad, Comunidad de Madrid, Spain; 10 Registro de Mortalidad, Consejería de Salud y Servicios Sociales, La Rioja, Spain; 11 Instituto Aragonés de Ciencias de la Salud, Zaragoza, Spain; 12 Instituto de Salud Pública de Navarra, Departamento de Salud, Gobierno de Navarra, Pamplona, Navarra Spain; 13 Unidad de Investigación en Análisis de la Mortalidad y Estadísticas Sanitarias, Universidad de Alicante, San Vicente del Raspeig, Spain; 14 Departamento de Medicina Preventiva y Salud Pública, Universidad de Santiago de Compostela, Santiago de Compostela, Spain; 15 Universitat Pompeu Fabra, Barcelona, Spain

**Keywords:** Disease mapping, Multilevel analysis, Geographical inequalities, Bayesian methods, Trends, Urban areas, Small areas, Mortality, Inequalities in mortality, Socioeconomic inequalities

## Abstract

**Background:**

In Spain, several ecological studies have analyzed trends in socioeconomic inequalities in mortality from all causes in urban areas over time. However, the results of these studies are quite heterogeneous finding, in general, that inequalities decreased, or remained stable. Therefore, the objectives of this study are: (1) to identify trends in geographical inequalities in all-cause mortality in the census tracts of 33 Spanish cities between the two periods 1996–1998 and 2005–2007; (2) to analyse trends in the relationship between these geographical inequalities and socioeconomic deprivation; and (3) to obtain an overall measure which summarises the relationship found in each one of the cities and to analyse its variation over time.

**Methods:**

Ecological study of trends with 2 cross-sectional cuts, corresponding to two periods of analysis: 1996–1998 and 2005–2007. Units of analysis were census tracts of the 33 Spanish cities. A deprivation index calculated for each census tracts in all cities was included as a covariate. A Bayesian hierarchical model was used to estimate smoothed Standardized Mortality Ratios (sSMR) by each census tract and period. The geographical distribution of these sSMR was represented using maps of septiles. In addition, two different Bayesian hierarchical models were used to measure the association between all-cause mortality and the deprivation index in each city and period, and by sex: (1) including the association as a fixed effect for each city; (2) including the association as random effects. In both models the data spatial structure can be controlled within each city. The association in each city was measured using relative risks (RR) and their 95 % credible intervals (95 % CI).

**Results:**

For most cities and in both sexes, mortality rates decline over time. For women, the mortality and deprivation patterns are similar in the first period, while in the second they are different for most cities. For men, RRs remain stable over time in 29 cities, in 3 diminish and in 1 increase. For women, in 30 cities, a non-significant change over time in RR is observed. However, in 4 cities RR diminishes. In overall terms, inequalities decrease (with a probability of 0.9) in both men (RR = 1.13, 95 % CI = 1.12–1.15 in the 1st period; RR = 1.11, 95 % CI = 1.09–1.13 in the 2nd period) and women (RR = 1.07, 95 % CI = 1.05–1.08 in the 1st period; RR = 1.04, 95 % CI = 1.02–1.06 in the 2nd period).

**Conclusions:**

In the future, it is important to conduct further trend studies, allowing to monitoring trends in socioeconomic inequalities in mortality and to identify (among other things) temporal factors that may influence these inequalities.

**Electronic supplementary material:**

The online version of this article (doi:10.1186/s12889-016-3190-y) contains supplementary material, which is available to authorized users.

## Background

Social inequalities in health, particularly in regard to mortality, are a relevant public health problem [1]. It is thus important to quantify them, determine their geographical patterns, and monitor them over time. Doing so makes it possible to provide evidence to governments and a diverse range of decision-makers and public administrators for the implementation of policies and interventions intended to reduce or erradicate these inequalities. Over the last decade there has been a proliferation of studies analysing socioeconomic inequalities in mortality taking geographical areas as the unit of analysis. There are at least three reasons for this proliferation. The first is the consideration that certain characteristics or attributes of an area of residence may be health determinants for the people living in them [2, 3]. The second is that studies of this type permit the identification of those geographical areas with unfavourable socioeconomic and health indicators [4]. The third is that using ecological data makes it more feasible to perform routine surveillance of health inequalities [5].

However, the inequalities in mortality may differ depending on whether one studies rural or urban areas; indeed inequalities tend to be greater in urban areas since often they include deprived and poor populations being concentrated in marginalized neighbourhoods and urban slums [5–7]. Also, the fact that the majority of the world’s population lives in urban areas means that the study of the processes acting in these areas is key to understanding the economic, cultural, political and health-related transformations which occur [4, 8, 9]. In Spain, numerous studies have reported the existence of socioeconomic inequalities in all-causes mortality in urban areas, and that areas with greater deprivation present higher mortality risk [10–16].

Various ecological studies have analysed time trends in socioeconomic inequalities in all-causes mortality in urban areas [17–23]. However, the findings of these studies are rather heterogeneous, not only between countries but also between urban areas of the same country. In the studies conducted outside Europe, an increase in inequalities over time was observed in Sydney [17], a slight reduction in New York [18] and that they remain stable in the Montreal metropolitan area [19]. Some studies have been carried out in the European context, specifically in Rome it was observed that inequalities were increasing with time, but stabilised near the end of the study period [20]. In Spain, trends in socioeconomic inequalities in mortality have been analysed in cities such as Sevilla [21], Cádiz [22], Barcelona and Madrid [23, 24], and in general it has been observed that inequalities either decline or remain stable over time.

This heterogeneity in the trends in inequalities in mortality at urban level may be due to, among other things, the different methodologies employed in the studies. Firstly, the cities analysed have different socioeconomic and epidemiological contexts which may mean that inequalites can either increase or decrease. Secondly, the different studies analyse geographical areas with different sizes and population densities. In this sense the effect of the so-called “Modifiable Areal Unit Problem” (MAUP) could make it difficult to compare findings between cities [25]. Thirdly, periods of time analysed differ between the different cities. Fourthly, some studies analyse premature mortality, while others analyse mortality for all ages. Finally, between the studies, different indicators and methods are used to determine levels of socioeconomic deprivation in small areas [26]. All this means that through these ecological studies of small areas it is difficult to obtain a clear view of the time trends of inequalities in mortality in urban areas.

With the aim of contributing more evidence on how these inequalities are evolving in Spain, the objectives of this study are: (1) to identify trends in geographical inequalities in all-cause mortality in the census tracts of 33 Spanish cities between the two periods 1996–1998 and 2005–2007; (2) to analyse trends in the relationship between these geographical inequalities and socioeconomic deprivation; and (3) to obtain an overall measure which summarises the relationship found in each one of the cities and to analyse its variation over time. This study forms part of the multicentric MEDEA project (http://www.proyectomedea.org) [23, 27, 28], which has made it possible to obtain data for the 33 cities in a relatively homogeneous manner, thus solving most of the problems described above. Moreover, in this study, apart from analysing each of the cities independently (the usual procedure), we have conducted a joint analysis by means of a multilevel random effects model, which allowed us to take account of the spatial structure of the data and obtain an overall result which summarises the behaviour of the cities as a whole (as if a metaanalysis had been performed).

## Methods

### Design, unit of analysis and study population

This is an ecological study of trends analysing comparing the two periods of time 1996–1998 and 2005–2007. The analysis units were the census tracts of 33 Spanish cities (Alicante, Almería, Avilés, Barcelona, Bilbao, Cádiz, Cartagena-La Unión, Castellón, Córdoba, Coruña, Ferrol, Gijón, Granada, Huelva, Jaén, Las Palmas de Gran Canaria (henceforth Las Palmas), Logroño, Lugo, Madrid, Málaga, Murcia, Ourense, Oviedo, Pamplona, Pontevedra, San Sebastián, Santa Cruz de Tenerife (henceforth Santa Cruz), Santiago, Sevilla, Valencia, Vigo, Vitoria-Gasteiz (henceforth Vitoria) and Zaragoza) as defined in the 2001 Housing and Population Census. These cities include 29.9 % of the 2001 population of Spain, are of varying sizes and are situated in different geographical regions of Spain (Additional file 1: Figure S1). The study population consisted of all persons resident in the 33 studied cities during the periods 1996–1998 and 2005–2007, except in the cases of Coruña, Ferrol, Lugo, Ourense, Pontevedra and Vigo where the study population consists of persons resident there during the year 1998 and the period 2005–2007.

### Sources of information

Data on mortality due to all causes, grouped by sex, census tract and period were obtained from the mortality registries of the corresponding Autonomous Communities. Census tracts were determined based on the residential address of the deceased, obtained from the death certificate or from the local census in each city. The proportion of deaths for which a census tract could not be assigned due to problems in geocoding the residential address varied from 0.02 % in Pamplona to 9.25 % in Ourense. Population data stratified by sex, age (in 5-year groups), census tract and period were obtained from the local census or from the Housing and Population Census, depending on availability in each of the cities (Instituto Nacional de Estadística). Finally, the socioeconomic indicators for census tracts in all the cities were obtained based on the 2001 Housing and Population Census.

### Socioeconomic deprivation index

An index of socioeconomic deprivation was included as a covariate, and was calculated for the census tracts of all the cities. To elaborate this index we used the five socioeconomic indicators finally proposed in the study by Domínguez-Berjón et al. [29]. These are: (1) Percentage of unemployment (people aged 16 years or over); (2) Percentage of manual workers (aged 16 years or over); (3) Percentage of temporary workers (aged 16 years or over); (4) Percentage of low educational level (people aged 16 years or over who are illiterate or who did not complete primary education); (5) Percentage of low educational level in young people (aged 16 to 29 years). These indicators were normalised (to have a mean of 0 and standard deviation of 1). The information contained in these five indicators was summarised in a deprivation index by means of a Principal Components Analysis, using the factor loadings of the first axis to weight each of the indicators. These factor loadings were 0.39, 0.46, 0.46, 0.47 and 0.45, respectively for each indicator. Finally, the deprivation index calculated explains an 88.1 % of the variance of the socioeconomic indicators and the Pearson correlation coefficients between the index and each indicator are respectively 0.70, 0.94, 0.85, 0.94 and 0.89. It should be noted that in the above-mentioned study by Domínguez-Berjón et al. [29] a separate deprivation was calculated for each city, consequently not permitting a comparison of the index values between areas of the different cities. With the aim of making the index values obtained comparable between cities, the analysis described above was conducted for all the cities jointly.

### Data analysis

Age standardised mortality rates (ASMR) were calculated by the direct method in five-year age groups, taking the 2001 Spanish population as the reference. The ASMR were calculated for each period, city and sex (Additional file 2: Table S1). Also, census tracts were grouped by tertiles of the deprivation index and ASMR obtained for census tracts below the first tertile (least deprivation) were compared with those above the third tertile (greatest deprivation). Specifically these ASMR were compared using absolute (differences) and relative (ratios) measures. All this was done for each period, city and sex (Tables 1 and 2).

**Table 1:** Age-standardised mortality rates (ASMR) among men, grouped by census tract tertiles of deprivation (1st tertile (T1) lower deprivation; 3rd tertile (T3) higher deprivation) and by city (33 cities in Spain)

	Period 1996–1998				Period 2005–2007				Reduction between periods 1996–1998 and 2005–2007			
CITIES	ASMR T1	ASMR T3	Ratio: T3/T1	Difference: T3-T1	ASMR T1	ASMR T3	Ratio: T3/T1	Difference: T3-T1	Difference for T1	Difference for T3	Percentage reduction for T1 (%)	Percentage reduction for T3 (%)
Alicante	1121	1275	1.14	154	815	1059	1.30	245	306	216	27	17
Almería	1208	1350	1.12	142	1020	1282	1.26	262	189	68	16	5
Avilés	1089	1299	1.19	210	1068	1152	1.08	84	21	147	2	11
Barcelona	1114	1438	1.29	324	945	1197	1.27	252	169	241	15	17
Bilbao	1296	1519	1.17	223	995	1186	1.19	191	301	333	23	22
Cádiz	1406	1534	1.09	128	1279	1286	1.00	6	126	248	9	16
Cartagena-La Unión	968	1484	1.53	516	1096	1246	1.14	150	−129	238	−13	16
Castellón	1398	1284	0.92	−114	1039	1184	1.14	145	359	100	26	8
Córdoba	1209	1394	1.15	184	1237	1146	0.93	−92	−28	248	−2	18
Coruña	1224	1396	1.14	173	918	1290	1.41	372	305	106	25	8
Ferrol	1230	1282	1.04	51	907	1279	1.41	372	324	3	26	0
Gijón	1253	1315	1.05	62	1015	1140	1.12	124	237	176	19	13
Granada	1288	1477	1.15	189	1036	1298	1.25	262	251	179	20	12
Huelva	1324	1595	1.20	271	1161	1462	1.26	301	162	132	12	8
Jaén	1019	1412	1.39	393	1183	1229	1.04	46	−164	183	−16	13
Las Palmas	1385	1408	1.02	22	968	1246	1.29	277	417	162	30	11
Logroño	1020	1231	1.21	211	1007	995	0.99	−12	13	236	1	19
Lugo	973	1193	1.23	220	1016	1027	1.01	11	−43	166	−4	14
Madrid	1082	1348	1.25	266	965	1164	1.21	200	117	184	11	14
Málaga	1203	1401	1.16	198	1136	1234	1.09	98	67	167	6	12
Murcia	1030	1213	1.18	184	983	1236	1.26	253	47	−23	5	−2
Ourense	1096	1154	1.05	59	1044	1046	1.00	2	51	108	5	9
Oviedo	1069	1418	1.33	350	900	1251	1.39	351	169	168	16	12
Pamplona	1077	1313	1.22	235	956	987	1.03	31	122	326	11	25
Pontevedra	1288	1250	0.97	−38	948	1047	1.10	99	340	203	26	16
San Sebastián	1265	1761	1.39	495	994	1299	1.31	305	272	462	21	26
Santa Cruz	1144	1296	1.13	152	886	1192	1.35	306	258	104	23	8
Santiago	1138	1579	1.39	441	937	827	0.88	−110	201	752	18	48
Sevilla	1274	1399	1.10	125	1181	1270	1.07	88	93	130	7	9
Valencia	1241	1492	1.20	251	1018	1133	1.11	115	224	359	18	24
Vigo	1093	1193	1.09	100	976	1108	1.14	132	117	85	11	7
Vitoria	1131	1239	1.09	107	931	1061	1.14	130	200	178	18	14
Zaragoza	1007	1197	1.19	190	1044	1186	1.14	141	−38	11	−4	1

ASMR: Mortality rate per 100,000 inhabitants, standardised for age by the direct method for the 2001 Spanish population

**Table 2:** Age-standardised mortality rates (ASMR) among women grouped by census tract tertiles of deprivation (1st tertile (T1) lower deprivation; 3rd tertile (T3) higher deprivation) and by city (33 cities in Spain)

	Period 1996–1998				Period 2005–2007				Reduction between periods 1996–1998 and 2005–2007			
CITIES	ASMR T1	ASMR T3	Ratio: T3/T1	Difference: T3-T1	ASMR T1	ASMR T3	Ratio: T3/T1	Difference: T3-T1	Difference for T1	Difference for T3	Percentage reduction for T1 (%)	Percentage reduction for T3 (%)
Alicante	639	662	1.04	23	502	562	1.12	60	136	100	21	15
Almería	704	825	1.17	121	603	714	1.18	111	101	111	14	13
Avilés	656	687	1.05	31	490	589	1.20	99	166	98	25	14
Barcelona	610	727	1.19	117	536	584	1.09	47	73	143	12	20
Bilbao	661	753	1.14	92	525	556	1.06	32	136	196	21	26
Cádiz	828	821	0.99	−7	700	730	1.04	30	128	91	15	11
Cartagena-La Unión	623	833	1.34	210	638	761	1.19	123	−16	72	−2	9
Castellón	812	713	0.88	−99	517	682	1.32	165	294	31	36	4
Córdoba	673	775	1.15	102	670	639	0.95	−31	3	136	0	18
Coruña	592	665	1.12	73	515	687	1.33	171	77	−21	13	−3
Ferrol	733	596	0.81	−137	639	666	1.04	27	95	−69	13	−12
Gijón	684	698	1.02	14	551	619	1.12	68	133	79	19	11
Granada	757	819	1.08	62	601	685	1.14	83	155	134	21	16
Huelva	743	775	1.04	33	593	731	1.23	137	149	45	20	6
Jaén	729	901	1.24	172	676	770	1.14	94	53	131	7	15
Las Palmas	773	821	1.06	48	587	727	1.24	140	186	94	24	11
Logroño	589	729	1.24	140	458	493	1.07	34	130	236	22	32
Lugo	568	611	1.08	43	505	629	1.25	125	64	−18	11	−3
Madrid	598	681	1.14	83	553	602	1.09	49	45	79	7	12
Málaga	737	809	1.10	72	657	707	1.08	50	80	103	11	13
Murcia	598	737	1.23	139	584	751	1.29	167	15	−14	2	−2
Ourense	578	646	1.12	69	507	602	1.19	95	70	44	12	7
Oviedo	582	676	1.16	94	521	599	1.15	78	61	77	11	11
Pamplona	554	702	1.27	148	455	610	1.34	155	99	92	18	13
Pontevedra	640	773	1.21	133	542	573	1.06	30	98	200	15	26
San Sebastián	660	790	1.20	130	517	597	1.15	80	143	193	22	24
Santa Cruz	634	709	1.12	76	564	651	1.15	86	70	59	11	8
Santiago	569	637	1.12	68	493	489	0.99	−4	76	148	13	23
Sevilla	754	787	1.04	32	687	673	0.98	−14	67	113	9	14
Valencia	649	844	1.30	196	555	674	1.22	120	94	170	14	20
Vigo	518	646	1.25	128	498	590	1.19	92	21	56	4	9
Vitoria	680	656	0.96	−24	512	487	0.95	−25	168	169	25	26
Zaragoza	550	634	1.15	84	595	618	1.04	22	−46	16	−8	3

ASMR: Mortality rate per 100,000 inhabitants, standardised for age by the direct method for the 2001 Spanish population

#### Model 1

Following the descriptive analysis in terms of ASMR, we analysed standardised mortality ratios (SMR). The SMR depend on the population since their variance is inversely proportional to the expected number of cases. Hence the areas with lower populations tend to present estimates with high variance [30]. In order to control this high variance in estimating the SMR we used the model proposed by Besag, York and Mollié (BYM) [30, 31], to calculate smoothed Standardised Mortality Ratios (sSMR). Specifically, we set up Model 1 to estimate sSMR values for the census tracts of the 33 cities, for each study period.

Model 1 is specified as follows. Let *O*_*ijt*_ be the observed number of deaths in census tract *i* (*i* = 1, …, *n*_*j*_) of city *j* (*j* = 1, …, 33) in period *t* (*t* = 1, 2), let *E*_*ijt*_ be the expected number of cases, and *θ*_*ijt*_ the relative risk. The expected number of cases in each census tract were calculated using the indirect method of standardisation [32], taking as reference for each city the all-causes mortality rates in the first period of the city in question, by five-year age groups.

In this model we take account of the spatial structure of the relative risks *θ*_*ijt*_, independently for each city *j* and period *t*. To do so, we include two random effects in the model, just as BYM propose. For the random spatial effect of each city *j* and period *t* we assume it follows an Intrinsic Conditional Autoregressive distribution (ICAR) [33] with a different variance *σ*_*S*_^2^_*jt*_ for each city and period. The second random effect *H*_*ijt*_, also known as the hetereogeneous (or non-spatial) effect assumes that the risks are distributed independently. For each city *j* and period *t*, we assigned an independent Normal distribution with mean zero and variance *σ*_*H*_^2^_*jt*_ to each *H*_*ijt*_. Vague Uniform distributions were assigned to the standard deviations (*σ*_*Sjt*_ and *σ*_*Hjt*_) of the random effects [34]. In this model the intercept (constant) is estimated independently for each city and period (*b*_1*jt*_). Hence, Model 1 may be formulated as follows:

$$ \begin{array}{c}{O}_{ijt}\sim Poisson\left({E}_{ijt}\cdot {\theta}_{ijt}\right),\kern0.5em i=1,\dots, {n}_j,\kern0.75em j=1,\dots, 33,\kern0.75em t=1,2\\ {}\\ {} \log \left({\theta}_{ijt}\right)={b}_{1jt}+{S}_{ijt}+{H}_{ijt}\\ {}\\ {}{b}_{1jt}\sim Uniform\left(-\infty, +\infty \right)\\ {}{S}_{ijt}\sim Intrinsic- CAR\left({\sigma}_{S_{jt}}^2\right)\\ {}{H}_{ijt}\sim Normal\left(0,{\sigma}_{H_{jt}}^2\right)\\ {}{\sigma}_{S_{jt}},{\sigma}_{H_{jt}}\sim Uniform\left(0,10\right)\end{array} $$

Note that this model is equivalent to fitting the model proposed by BYM with random effects, for each city and period. Thus, with this model we obtain the sSMR values for census tract *i*, city *j* and period *t* via the formula: exp[*b*_1*jt*_ + *S*_*ijt*_ + *H*_*ijt*_] ⋅ 100.

**Fig. 1:**
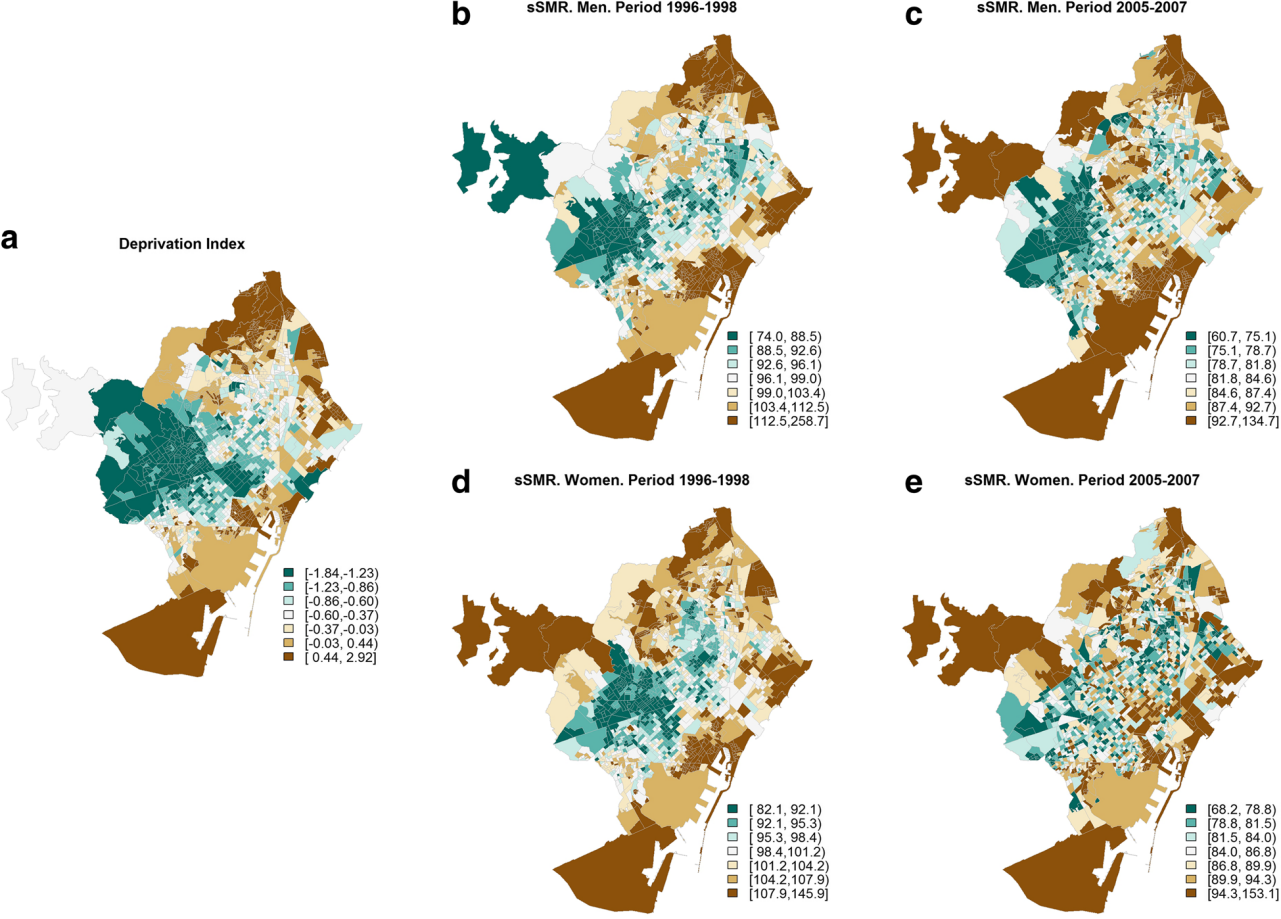
Distribution of deprivation index (**a**) and of the smoothed Standardised Mortality Ratios (sSMR) (**b**-**e**) for all-cause mortality, by period (1996–1998 and 2005–2007) and by sex in the city of Barcelona. Green areas represent less socioeconomic deprivation and lower sSMR values. Brown areas represent greater socioeconomic deprivation and higher sSMR values

The geographical distribution of the sSMR values in each of the cities and for each of the periods 1996–1998 (*t* = 1) and 2005–2006 (*t* = 2) has been represented in the form of maps of septiles. Together with these maps of mortality, we provide the geographical distribution of the deprivation index in each of the cities, again using septile maps. This makes it possible to check visually whether the spatial distribution of mortality has varied over time, and whether or not it is similar to the spatial distribution of deprivation. Figure 1 presents these maps for the city of Barcelona, as an example. The maps for the other cities may be consulted in the Additional file 3.

#### Model 2

Model 2 is an ecological regression in which the aim is to study the association between mortality and deprivation in each of the periods, as well as their evolution over time. In order to make the risks *θ*_*ijt*_ comparable between the different census tracts, cities and periods, the expected cases *E*_*ijt*_ have been calculated by the indirect method taking the 2001 all-cause mortality rates for Spain as reference. Thus, Model 2 is an extension of Model 1 in which we incorporate (as fixed effects):

1. A covariate *X*_*ij*_, the index of socioeconomic deprivation.
2. The study periods, by adding a categorical variable *T*_2*t*_ to the model, which takes the value 0 for the period 1996–1998 (*t* = 1) and 1 for 2005–2007 (*t* = 2).
3. The interaction of the variables specified in the above two points, in order to be able to study the relationship between socioeconomic deprivation index and mortality in each period, and also to quantify whether there has been any change in this relationship.

Finally, Model 2 is specified as follows:

$$ \begin{array}{c}{O}_{ijt}\sim Poisson\left({E}_{ijt}\cdot {\theta}_{ijt}\right),\kern0.5em i=1,\dots, {n}_j,\kern0.75em j=1,\dots, 33,\kern0.75em t=1,2\\ {}\\ {} \log \left({\theta}_{ijt}\right)={b}_{1j}+{b}_{2j}\cdot {X}_{ij}+{b}_{3j}\cdot {T}_{2t}+{b}_{4j}\cdot {X}_{ij}\cdot {T}_{2t}+{S}_{ijt}+{H}_{ijt}\\ {}\\ {}{b}_{kj}\sim Uniform\left(-\infty, +\infty \right),\kern0.5em k=1,\dots, 4\\ {}{S}_{ijt}\sim Intrinsic- CAR\left({\sigma}_{S_{jt}}^2\right)\\ {}{H}_{ijt}\sim Normal\left(0,{\sigma}_{H_{jt}}^2\right)\\ {}{\sigma}_{S_{jt}},{\sigma}_{H_{jt}}\sim Uniform\left(0,10\right)\end{array} $$

As may be seen, both the model intercepts (*b*_1*j*_) and parameters (or coefficients) associated with the different variables of the model and their interactions (*b*_*kj*_, *k* = 2, …, 4), differ and are independent for each city *j*. Therefore, this model is equivalent to fitting separate ecological regressions (with the random effects proposed in the BYM model) for each one of the cities.

**Fig. 2:**
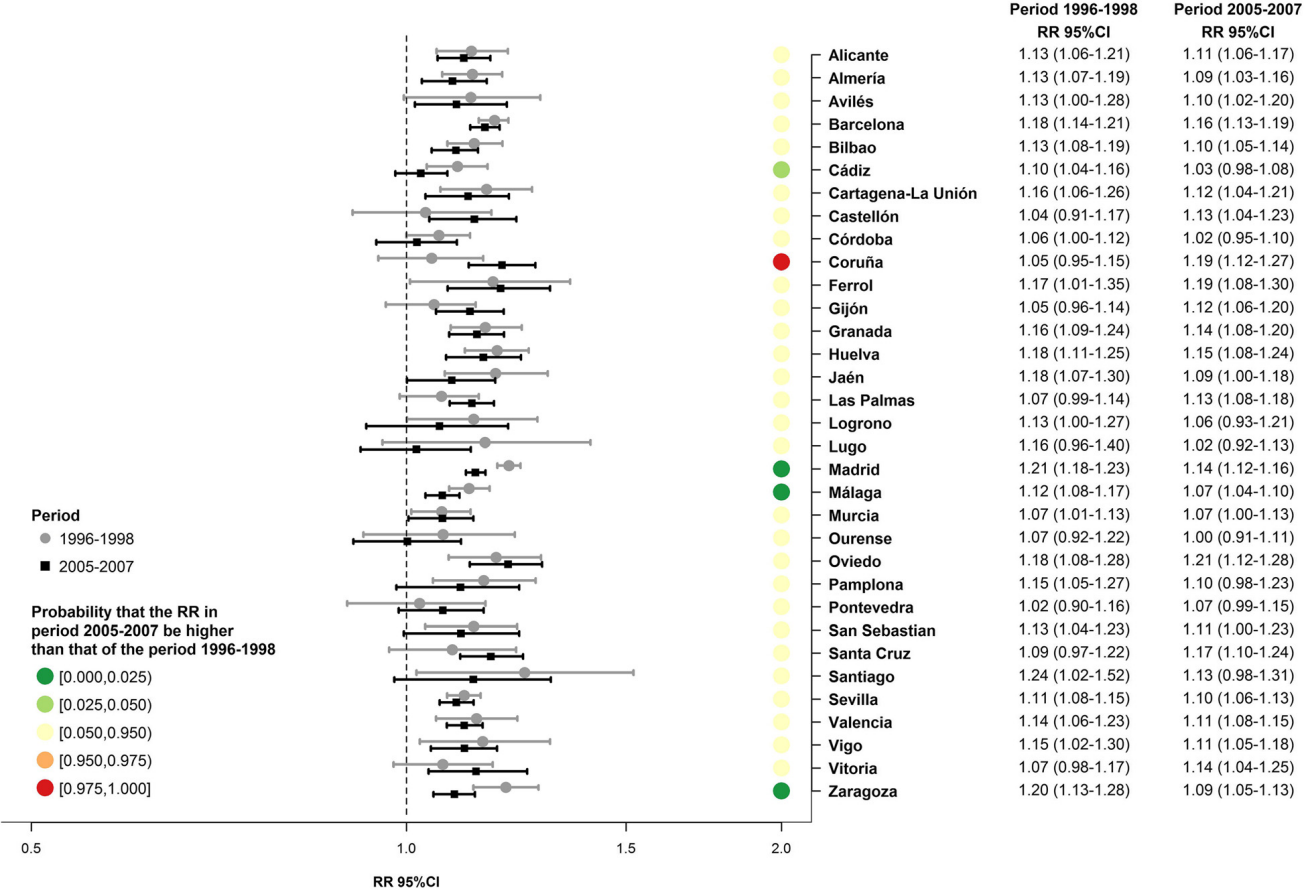
Association between all-cause mortality among men and the deprivation index, as estimated by Model 2. Relative risk (RR) and its 95 % credible interval (95 % CI) by period (1996–1998 and 2005–2007) in 33 Spanish cities

**Fig. 3:**
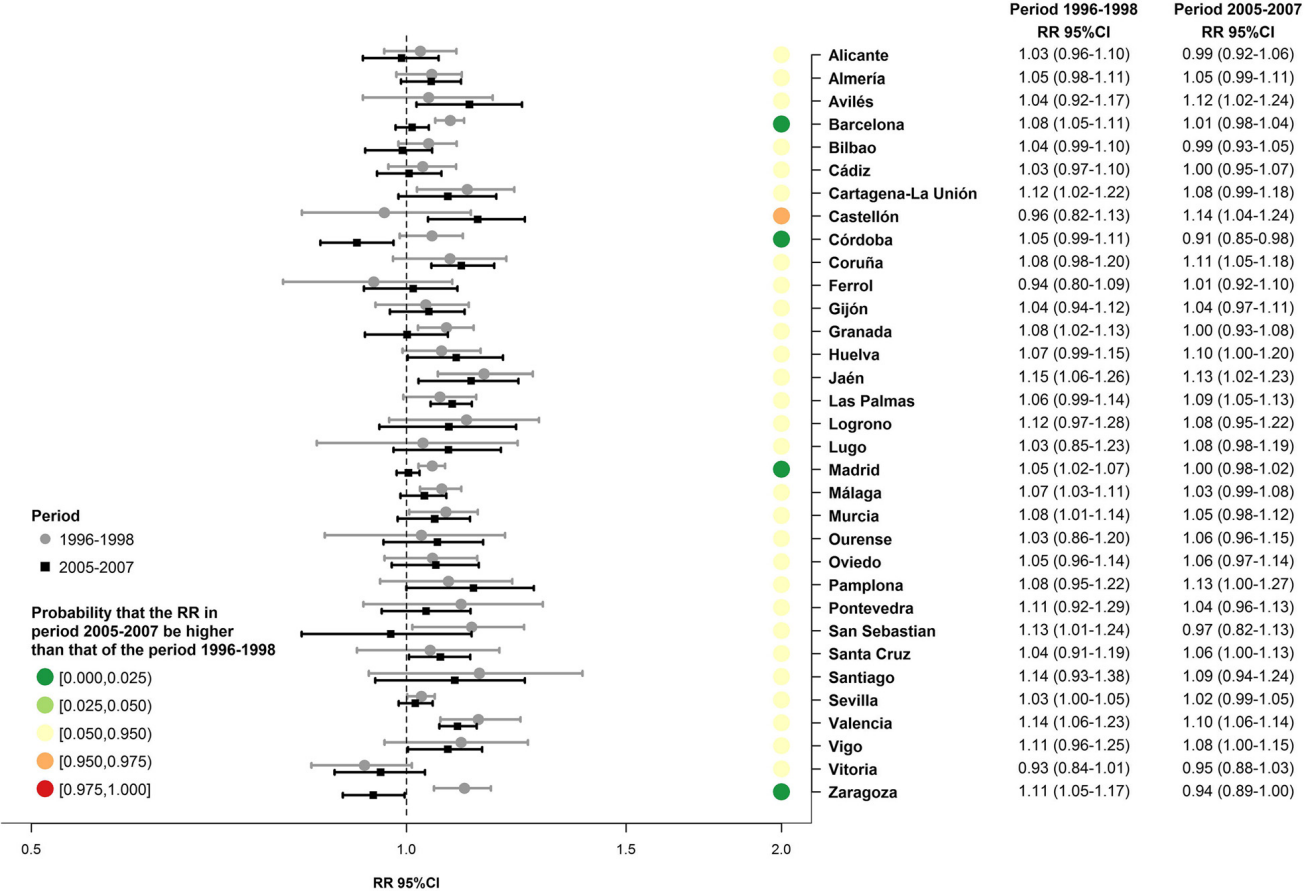
Association between all-cause mortality among women and the deprivation index, as estimated by Model 2. Relative risk (RR) and its 95 % credible interval (95 % CI) by period (1996–1998 and 2005–2007) in 33 Spanish cities

The main results of this model are presented in Fig. 2 (for men) and Fig. 3 (for women). The relative risk (RR) of dying, for each unit increment of the deprivation index during the period 1996–1998, for each city *j*, is given by exp(*b*_2*j*_). For the period 2005–2007, the corresponding RR is given by exp(*b*_2*j*_ + *b*_4*j*_). Finally, to see whether the RR change from one period to the other, we calculated the probability that the RR in period 2005–2007 be higher than that of the period 1996–1998, i.e. Pr(exp(*b*_2*j*_ + *b*_4*j*_) > exp(*b*_2*j*_)) = Pr(*b*_4*j*_ > 0). In what follows, we will refer to this probability as Pr(*Δ*RR). This probability has been categorized in 5 groups [0.000, 0.025), [0.025, 0.050), [0.050, 0.950), [0.950, 0.975) and [0.975, 1.000]. We consider that the RR change significantly over time if Pr(*Δ*RR) belongs to the first or the fifth group; indicating a significantly decrease in the fist case and an increase in the second. Note that this is equivalent to asses whether the 95 % credible interval of *b*_4*j*_ (coefficient associated to the interaction term between deprivation and periods) excludes 0.

#### Model 3

This model is based on Model 2, but here the intercepts (*b*_1*j*_) and parameters associated with the different model variables and their interactions (*b*_*kj*_, *k* = 2, …, 4), are considered to be random instead of fixed effects. Specifically we consider that, for the whole set of cities, each one of the 4 parameters follows a normal distribution with unknown mean and variance. Model 3 is conceptually different from Model 2 since it considers that the cities in the study come from a random sample of the large cities in Spain, and as such, we will attempt to determine the association, object of the study, in the population to which they belong. Thus Model 3, when estimating the association between mortality and deprivation index (second period) takes into account that the regression coefficients in the different cities have a common distribution and behaviour, and thus mutually share information. Moreover, this model may be considered as multi-level, in which the first level are the census tracts, and the second level the cities. The model is specified as follows:

$$ \begin{array}{c}{O}_{ijt}\sim Poisson\left({E}_{ijt}\cdot {\theta}_{ijt}\right),\kern0.5em i=1,\dots, {n}_j,\kern0.75em j=1,\dots, 33,\kern0.75em t=1,2\\ {}\\ {} \log \left({\theta}_{ijt}\right)={b}_{1j}+{b}_{2j}\cdot {X}_{ij}+{b}_{3j}\cdot {T}_{2t}+{b}_{4j}\cdot {X}_{ij}\cdot {T}_{2t}+{S}_{ijt}+{H}_{ijt}\\ {}\\ {}{b}_{kj}\sim Norma{l}_J\left({\beta}_k,{\sigma}_{b_k}^2\right),\kern0.5em k=1,\dots, 4\\ {}{S}_{ijt}\sim Intrinsic- CAR\left({\sigma}_{S_{jt}}^2\right)\\ {}{H}_{ijt}\sim Norma l\left(0,{\sigma}_{H_{jt}}^2\right)\\ {}{\beta}_k\sim Uniform\left(-\infty, +\infty \right),\kern0.5em k=1,\dots, 4\\ {}{\sigma}_{b_k},{\sigma}_{S_{jt}},{\sigma}_{H_{jt}}\sim Uniform\left(0,10\right),\kern0.5em k=1,\dots, 4\end{array} $$

The main results of this model are presented in Additional file 4: Figure S2 (for men) and Additional file 5: Figure S3 (for women). These figures show the same RR and probabilities described above for Model 2. However, Model 3 in addition allows us to estimate a global RR which summarises the association found in each one of the cities. This global RR in the period 1996–1998 is given by the expression exp(*β*_2_). Similarly, the global RR for the period 2005–2007 is estimated by exp(*β*_2_ + *β*_4_). Finally, the probability that the global RR for the period 2005–2007 is higher than the global RR for the period 1996–1998 is calculated via the expression Pr(*β*_4_ > 0). These probabilities have been categorized and interpreted as in the model 2.

All models were fitted using a completely Bayesian approach. The posterior distributions were obtained using Markov Chain based Monte Carlo methods via WinBUGS and R [35, 36]. Convergence was assessed by the Gelman-Rubin convergence diagnosis $\left(\widehat{R}\right)$ and calculation of the effective number of independent values in the chains (*n*_*eff*_). The criteria for convergence was $\widehat{R}<1,1$ and *n*_*eff*_ > 100 for all model parameters [37]. Point estimates of the parameters were obtained using posterior means. As mentioned previously, some of the estimates are accompanied by their respective 95 % credible intervals (95 % CI).

## Results

Additional file 2: Table S1 shows the numbers of census tracts in each city. The number varies from 57 in Pontevedra to 2358 in Madrid. This table also includes the numbers of deaths, population at risk, and the ASMR per 100,000 inhabitants by period and sex. These rates tend to decline in all the cities and in both sexes, with the exception of Zaragoza where rise in both sexes.

For the majority of cities and in both sexes, the ASMR fall, both in census tracts with less deprivation (first tertile of deprivation), and in those with more deprivation (third deprivation tertile) (Tables 1 and 2). In general, the variations over time of the absolute and relative differences in mortality (between census tracts with less and with more deprivation) go in the same direction. Thus, when the absolute differences increase, so do the relative differences, and similarly, when absolute differences decrease so do the relative differences. In both men and women, we observe that in 20 of the 33 cities the absolute differences decrease over time, while the relative differences decrease in 17 of them. These decreases are due, in the majority of cases, to a greater reduction in mortality in the census tracts with more deprivation, in comparison to those with less deprivation.

In men, all-cause mortality presents a similar geographical pattern to socioeconomic deprivation for the majority of cities and both periods (Additional file 3). In women in the first period we also observe this similarity in the majority of the cities. However, in the second period these spatial patterns become different in most of the cities studied. For example, Fig. 1 presents the geographical distributions of the deprivation index and of the sSMR for the city of Barcelona in the two study periods (1996–1998 and 2005–2007) and by sex. In this figure we may observe how, in men, the geographical distribution of the deprivation index (Fig. 1a) is similar to the geographical distribution of the sSMR in both periods (Fig. 1b and c). The women follow the same pattern, except in the second period where it can clearly be seen how the sSMR have a rather heterogeneous spatial pattern, which is different from that of deprivation (Fig. 1e).

Figure 2 presents the association between the deprivation index and all-cause mortality in men, by city and period, obtained using Model 2. The RR are over 1 in the two periods in all cities, and these RR are significant (95 % CI does not include 1) in 24 cities in the first period and in 25 in the second. Looking at the time trend in the RR (i.e. reflected by the probability Pr(*Δ*RR)), represented using coloured dots in the figure, we see that in 29 cities there is no significant change over time in the RR (Pr(*Δ*RR) ∈ [0.025, 0.975)). However, in 3 cities (Madrid, Málaga and Zaragoza) the RR decrease with time and in 1 (Coruña) the RR increase. In regard to women (Fig. 3), the RR are over 1 in the two periods in the majority of the cities, and significant in 11 cities in the first period and in 10 in the second. In contrast, only in the city of Córdoba do we find a RR significantly under 1 (RR = 0.91, 95 % CI = (0.85–0.98). Turning to the trends over time in the RR represented by Pr(*Δ*RR), we see that in 29 cities there is no significant evolution in the RR (Pr(*Δ*RR) ∈ [0.025, 0.975)). However, in 4 cities (Barcelona, Córdoba, Madrid and Zaragoza) the RR decrease with time. In general the RR for women are lower than those for men in both periods.

Additional file 4: Figure S2, like Fig. 2, presents the association between the deprivation index and all-cause mortality among men, by city and period, obtained using Model 3. In general this model provides much more consistent associations between the different cities than Model 2, since in all the cities and in both periods we obtained RRs significantly greater than 1. Also, it may be seen that in 4 cities (Cádiz, Madrid, Málaga and Zaragoza) the RR decrease with time and the rest of the cities remain stable (Pr(*Δ*RR) ∈ [0.025, 0.975)). If we look at the RR obtained using Model 3 in women (Additional file 5: Figure S3), we see that in the first period they are significantly greater than 1 in all the cities except Vitoria. In the second period, the majority of cities have an RR greater than 1, being significantly so in 19 of them. Also, we may observe that in 4 cities (Barcelona, Córdoba, Madrid and Zaragoza) the RR decrease with time and the rest of the cities remain stable (Pr(*Δ*RR) ∈ [0.025, 0.975)). Via Model 3 we may also observe that, in general, RR for women are lower than those for men in both periods.

Finally, through Model 3 we calculated a global RR (RRg) which summarises the association of all the cities. This RRg was calculated for each period and sex (Additional file 4: Figure S2 and Additional file 5: Figure S3). In men, the RRg is significantly greater than 1 both in the first period (RRg = 1.13, 95 % CI = 1.12–1.15), and in the second period (RRg = 1.11, 95 % CI = 1.09–1.13). Moreover, the probability that RRg in the second period be greater than that of the first period is very low (under 0.025) which implies that the RRg have fallen with time. Among women the behaviour of the RRg is similar to that of men, although the associations found are weaker. Specifically, the RRg for the first period is 1.07 (95 % CI = 1.05–1.08) and in the second period is 1.04 (95 % CI = 1.02–1.06), with a probability that the second be greater than the first of under 0.025.

## Discussion

### Main findings

This study shows that inequalities of all-causes mortality in terms of socioeconomic deprivation persist in the two periods, and in men and women. In general, these inequalities are greater among men than among women. Finally, although with a certain variability between the cities, overall these inequalities decrease over time in both sexes.

In the majority of Spanish cities where inequalities in mortality have been studied it has been observed that they remain stable or decrease [21, 23, 24, 38, 39]. In this study, we corroborate the findings of those studies, as only in 1 city among men, we have seen that inequalities increased significantly. Finally, the joint estimate for all the cities using the random effects model allows us to conclude that overall inequalities decrease in both sexes. Moreover, our results point out in the same direction as other studies analyzing small areas at the urban level in other countries [18–20].

### Possible causes of the decrease in inequalities

Various studies attribute the decrease in inequalities in mortality to trends over time in people’s lifestyles (for example smoking, alcohol consumption and diet) depending on their socioeconomic level [1, 40]. In women the decline in inequalities may be due, above all, to the fact that older women of higher socioeconomic level adopted lifestyles that had previously been adopted by men and, therefore, their mortality rates decreased less than those of women of lower socioeconomic levels [41]. However, these lifestyles ought to be considered as only part of the trends in inequalities, since these are influenced by poor material conditions of everyday life and by limited access to fundamental determinants of health [14, 42].

On the other hand, in recent years there has been a decline in the numbers of AIDS deaths, mainly due to the introduction of antiretroviral therapy, and in deaths due to drug consumption. These deaths mainly affected groups of lower socioeconomic level [43, 44].

During the period studied (1996–2007), in Spain the percentage of foreign population has risen considerably, from 1.37 % in 1996 to 10 % in 2007, according to the National Institute of Statistics. There is abundant evidence to show that immigrants, particularly those arriving most recently, have better average health than the native population [45] and in addition are young and therefore with low mortality. A high percentage of the immigrant population resides in areas of more deprivation, which could lead to a reduction in the observed cases of death in these areas. Therefore, we may propose the hypothesis that the reduction in inequalities could be explained in part by this phenomenon [19, 46].

The periods analysed in this study are previous to the economic crisis which Spain is currently suffering. In fact, the period 1996–2007 was characterised by being a period of economic expansion which affected all strata of society. Thus, for example, the unemployment rate in 1996 was 20.5 %, and decreased steadily to 7.9 % in 2007, almost three times lower, and the lowest rate in Spain’s history. Also, during the study period there was a gradual improvement in the national health system, and specifically in the access to health services and in quality of health care. This improvement, among other things, has led to a reduction in deaths from cardiovascular diseases [47].

### Strengths and limitations

The main limitation of this study is the utilisation of a single deprivation index in the two studied periods, since this implies assuming that the distribution of deprivation is constant over time. However, we believe that the distribution of socioeconomic deprivation at the level of small areas has not suffered important variations, since the process which provokes changes in area deprivation is slow [48, 49]. One of the strengths of this study is that the deprivation index used allows comparisons between the small areas of different cities. Moreover, the conceptual domains considered by this index match with those commonly used in Spain for studies of similar characteristics [50]. In addition, it is the first to analyse trends in socioeconomic inequalities in all-cause mortality in a large number of Spanish cites with heterogeneous socioeconomic and epidemiological contexts, thus providing a global view of the behaviour of mortality in urban areas, while also contributing to improve consistency of the findings. Moreover, in all the cities small areas (census tracts) have been used, which means a high level of spatial disaggregation of the results. Among other advantages, these areas permit representation on maps of high “resolution”, capable of reflecting spatial patterns that using larger areas, such as neighbourhoods, would conceal. Therefore, they partly avoid MAUP [51]. Another strength of the study is that, as far as we know, it is the first to employ a multilevel random effects model (Model 3), in which the first level corresponds to the census tracts and the second level to the cities. This model, we believe, has certain advantages over conducting an independent analysis for each city (Model 2). In the first place, it takes account of the within- and between-city variability, thus yielding more robust estimates than with Model 2. In second place, it has allowed us to obtain an overall association which summarises the association between mortality and deprivation in all the cities, i.e. it is as if we had conducted an independent study in each one of the cities, and subsequently a meta-analysis of them to obtain an overall association. However, in our case we analysed all the data together, unlike a meta-analysis which would require two steps. Thus, this model has allowed us to provide an overall result which contributes clear evidence of how inequalities are evolving in the large cities in Spain. This is an important advantage, if we remember that the previous evidence was fairly heterogeneous.

## Conclusions

Although socioeconomic inequalities in all-cause mortality remain stable or decrease in the majority of the cities studied and in both sexes, it should be noted that these inequalities still persist significantly in many of them. In order to maintain (or even accentuate) this reduction it is important that policies and programmes aiming to reduce them pay attention to the “causes of the causes” of inequalities in health, in other words to the living and working conditions of the population, apart from contextual risk factors specific to the particular area of residence. Moreover, it is important that they take into account that these risk factors or determinants may differ between cities [52]. In this sense, studies like ours are particularly appropriate, since they compare different cities and permit the identification, with a high level of disaggregation, of those areas most susceptible to intervention. In the future it is important to continue performing studies of trends in terms of causes of death as they allow us to monitor trends in socioeconomic inequalities in mortality and to detect (among other things) temporal factors which may influence these inequalities, such as the economic crisis which since 2008 has affected (mainly) Southern Europe, and hence the cities here analysed.

## Abbreviations

AIDS, acquired immune deficiency syndrome; ASMR, age standardised mortality rate; BYM, Besag, York and Mollié; CI, credible interval; ICAR, Intrinsic Conditional Autoregressive; MAUP, Modifiable Areal Unit Problem; RR, relative risk; RRg, global RR; sSMR, smoothed Standardized Mortality Ratio; T1, 1st tertile; T3, 3rd tertile

## Additional files

Additional file 1: Figure S1. Geographical distribution of the 33 cities studied. (DOC 368 kb) Additional file 2: Table S1. Numbers of census tracts, numbers of deaths for all causes, population at risk and age-standardised mortality rates (ASMR) by sex, period (1996–1998 and 2005–2007) and city (33 cities in Spain). Shows the numbers of census tracts in each city. The number varies from 57 in Pontevedra to 2358 in Madrid. This table also includes the numbers of deaths, population at risk, and the ASMR per 100,000 inhabitants by period and sex. These rates tend to decline in all the cities and in both sexes, with the exception of Zaragoza where rise in both sexes. (DOC 133 kb) Additional file 3: Atlas of mortality in 33 Spanish cities (periods 1996–1998 and 2005–2007). The geographical distribution of the sSMR values in each of the cities and for each of the periods 1996–1998 (*t* = 1) and 2005–2006 (*t* = 2) has been represented in the form of maps of septiles. Together with these maps of mortality, we provide the geographical distribution of the deprivation index in each of the cities, again using septile maps. This makes it possible to check visually whether the spatial distribution of mortality has varied over time, and whether or not it is similar to the spatial distribution of deprivation. (PDF 42436 kb) Additional file 4: Figure S2. Association between all-cause mortality among men and the deprivation index, as estimated by Model 3. Relative risk (RR) and its 95 % credible interval (95 % CI) by period (1996–1998 and 2005–2007) in 33 Spanish cities. (DOC 1133 kb) Additional file 5: Figure S3. Association between all-cause mortality among women and the deprivation index, as estimated by Model 3. Relative risk (RR) and its 95 % credible interval (95 % CI) by period (1996–1998 and 2005–2007) in 33 Spanish cities. (DOC 1183 kb)
