# Supplementary figures and images for: Trends in socioeconomic inequalities in mortality in small areas of 33 Spanish cities
Source: BMC Public Health. 2016 Jul 29;16:663. doi: 10.1186/s12889-016-3190-y (PMC4966571; doi:10.1186/s12889-016-3190-y)

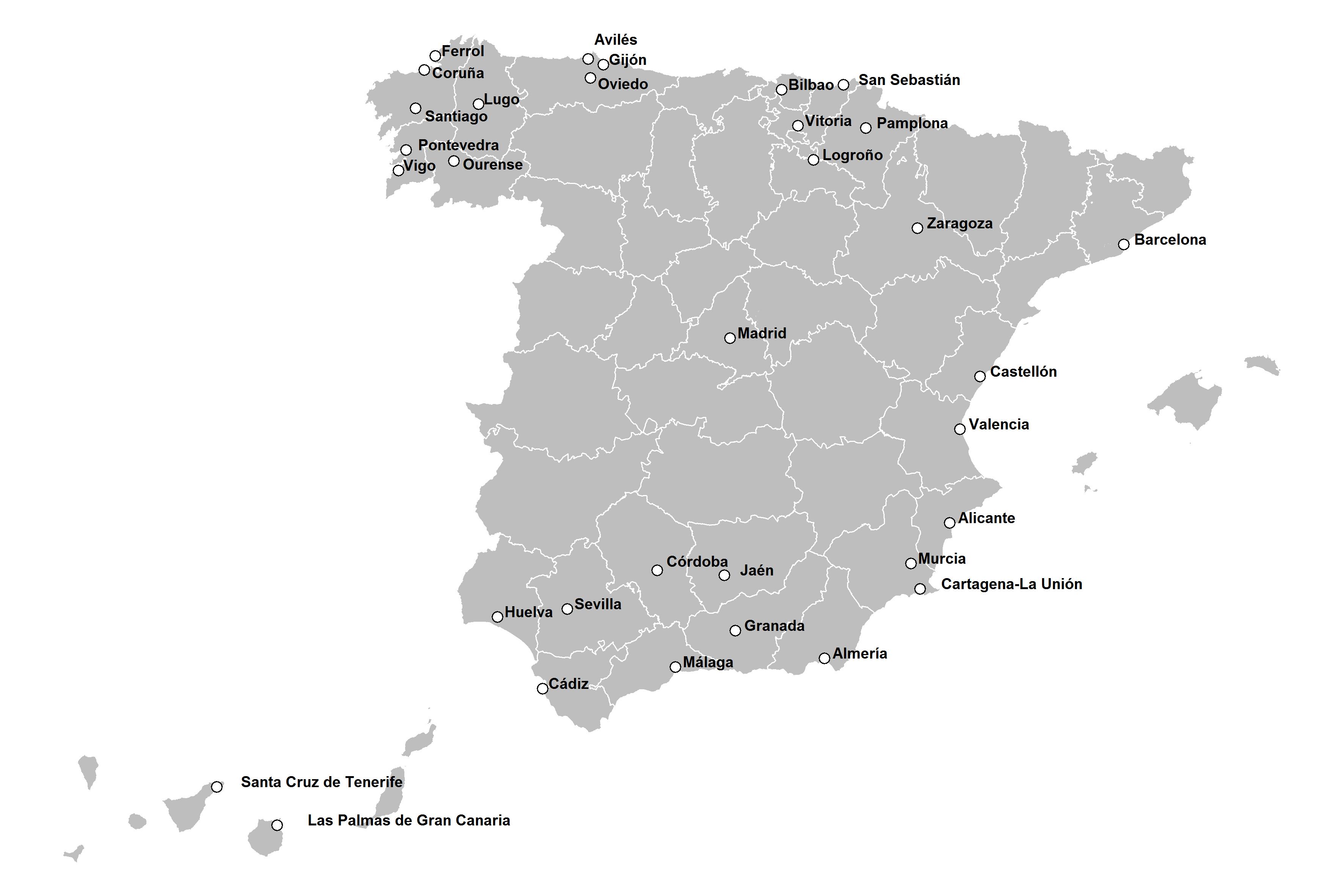

Supplement: Additional file 1: Figure S1. — Geographical distribution of the 33 cities studied. (DOC 368 kb) [file 12889_2016_3190_MOESM1_ESM.doc]

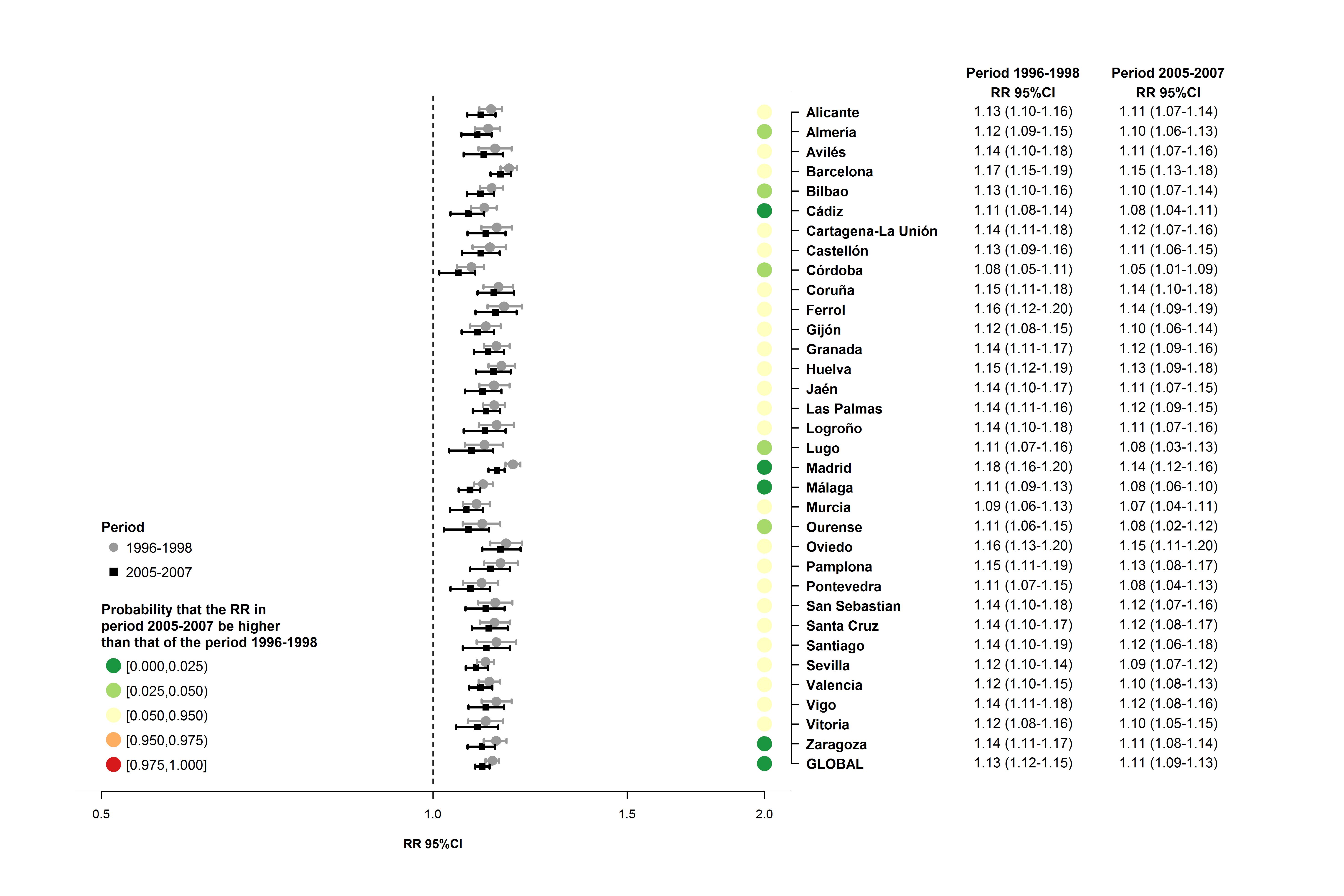

Supplement: Additional file 4: Figure S2. — Association between all-cause mortality among men and the deprivation index, as estimated by Model 3. Relative risk (RR) and its 95 % credible interval (95 % CI) by period (1996–1998 and 2005–2007) in 33 Spanish cities. (DOC 1133 kb) [file 12889_2016_3190_MOESM4_ESM.doc]

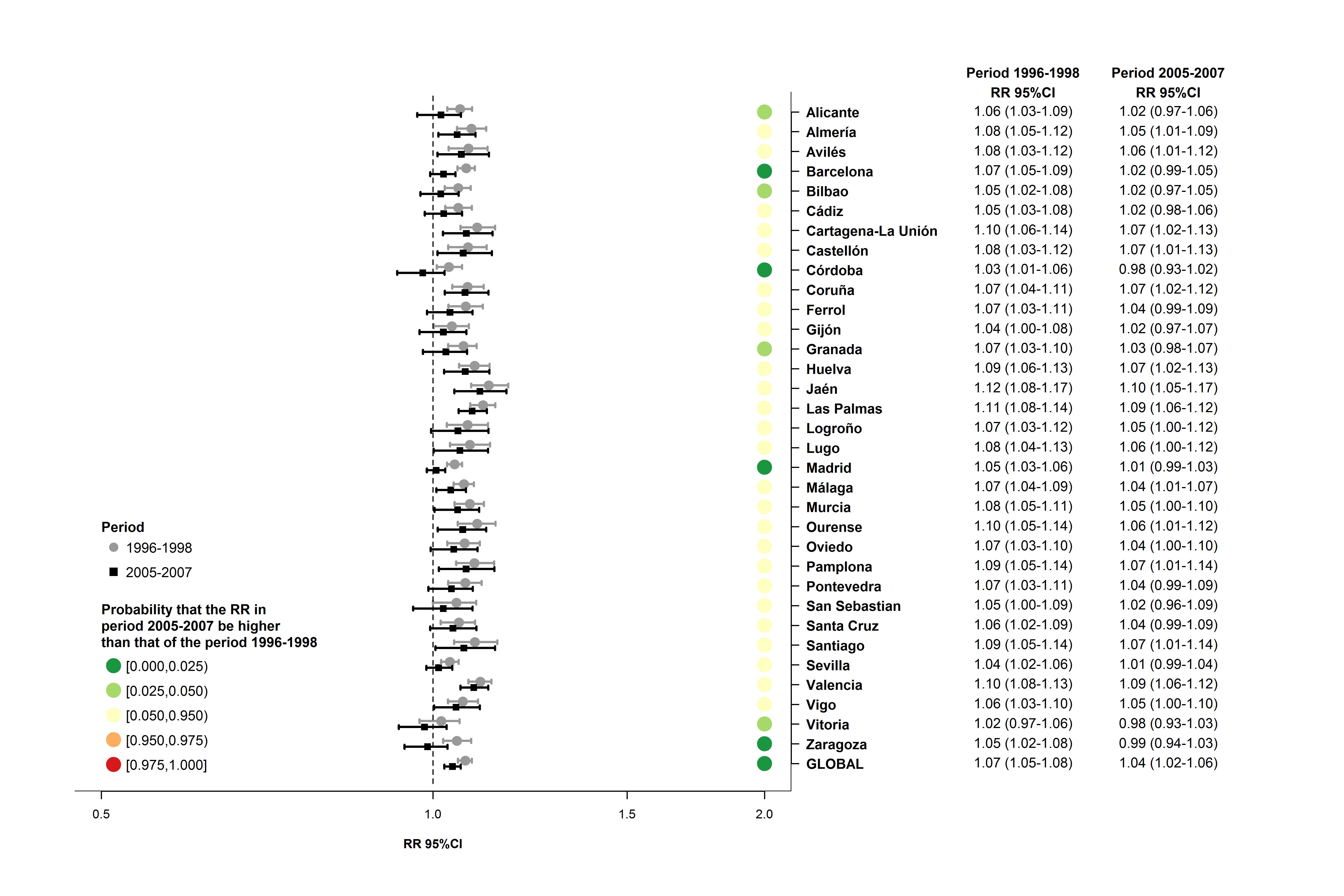

Supplement: Additional file 5: Figure S3. — Association between all-cause mortality among women and the deprivation index, as estimated by Model 3. Relative risk (RR) and its 95 % credible interval (95 % CI) by period (1996–1998 and 2005–2007) in 33 Spanish cities. (DOC 1183 kb) [file 12889_2016_3190_MOESM5_ESM.doc]
