# Supplementary material for: Trends in socioeconomic inequalities in mortality in small areas of 33 Spanish cities
Source: BMC Public Health. 2016 Jul 29;16:663. doi: 10.1186/s12889-016-3190-y (PMC4966571; doi:10.1186/s12889-016-3190-y)
Supplement: Additional file 2: Table S1. — Numbers of census tracts, numbers of deaths for all causes, population at risk and age-standardised mortality rates (ASMR) by sex, period (1996–1998 and 2005–2007) and city (33 cities in Spain). Shows the numbers of census tracts in each city. The number varies from 57 in Pontevedra to 2358 in Madrid. This table also includes the numbers of deaths, population at risk, and the ASMR per 100,000 inhabitants by period and sex. These rates tend to decline in all the cities and in both sexes, with the exception of Zaragoza where rise in both sexes. (DOC 133 kb) [file 12889_2016_3190_MOESM2_ESM.doc]

**Online Table 1.** Numbers of census tracts, socioeconomic deprivation index percentiles, numbers of deaths for all causes, population at risk and age-standardised mortality rates (ASMR) by sex, period (1996-1998 and 2005-2007) and city (33 cities in Spain).

|  |  |  |  |  | **Men** | | | | | | **Women** | | | | | |
| --- | --- | --- | --- | --- | --- | --- | --- | --- | --- | --- | --- | --- | --- | --- | --- | --- |
|  |  | **Deprivation Index** | | | **Period 1996-1998** | | | **Period 2005-2007** | | | **Period 1996-1998** | | | **Period 2005-2007** | | |
| **CITIES** | **Census tracts** | **P_25_** | **P_50_** | **P_75_** | **Deaths** | **Population**  **at risk** | **ASMR** | **Deaths** | **Population**  **at risk** | **ASMR** | **Deaths** | **Population**  **at risk** | **ASMR** | **Deaths** | **Population**  **at risk** | **ASMR** |
| **Alicante** | 215 | -0.42 | 0.22 | 0.77 | 3463 | 394646 | 1224 | 3654 | 468850 | 966 | 2967 | 428007 | 670 | 3316 | 495634 | 538 |
| **Almería** | 118 | -0.34 | 0.57 | 1.31 | 1841 | 246684 | 1292 | 2021 | 257514 | 1199 | 1738 | 262227 | 790 | 1818 | 272613 | 666 |
| **Avilés** | 72 | -0.16 | 0.41 | 1.08 | 1203 | 123164 | 1343 | 1310 | 120147 | 1118 | 972 | 132674 | 705 | 1101 | 130566 | 557 |
| **Barcelona** | 1491 | -0.93 | -0.49 | 0.08 | 24202 | 2148417 | 1208 | 23269 | 2277866 | 1016 | 23735 | 2441846 | 643 | 24256 | 2526810 | 544 |
| **Bilbao** | 288 | -0.83 | -0.25 | 0.37 | 5502 | 511188 | 1369 | 5544 | 497274 | 1074 | 5033 | 565437 | 700 | 5244 | 556065 | 539 |
| **Cádiz** | 111 | -0.03 | 1.00 | 1.86 | 1821 | 210204 | 1494 | 1899 | 191085 | 1317 | 1656 | 226647 | 812 | 1808 | 208641 | 733 |
| **Cartagena-La Unión** | 146 | 0.25 | 1.09 | 1.52 | 2503 | 281151 | 1396 | 2534 | 321225 | 1182 | 2114 | 288297 | 807 | 2309 | 317631 | 717 |
| **Castellón** | 95 | -0.38 | -0.02 | 0.43 | 1870 | 200073 | 1301 | 1962 | 253271 | 1074 | 1557 | 211077 | 749 | 1702 | 258918 | 598 |
| **Córdoba** | 224 | -0.13 | 0.76 | 1.44 | 3555 | 441513 | 1356 | 3773 | 461973 | 1165 | 3198 | 475515 | 750 | 3632 | 497103 | 648 |
| **Coruña** | 181 | -0.79 | -0.27 | 0.03 | 1083 | 113548 | 1205 | 3358 | 340771 | 1015 | 954 | 129586 | 608 | 3329 | 390286 | 556 |
| **Ferrol** | 69 | -0.44 | 0.02 | 0.55 | 396 | 38559 | 1193 | 1262 | 106653 | 1108 | 374 | 43986 | 651 | 1319 | 122082 | 630 |
| **Gijón** | 191 | -0.35 | 0.28 | 0.70 | 4175 | 377726 | 1323 | 4421 | 390207 | 1098 | 3702 | 416770 | 699 | 4171 | 432233 | 592 |
| **Granada** | 181 | -0.67 | -0.18 | 0.69 | 3147 | 348435 | 1412 | 3199 | 330957 | 1152 | 2954 | 397005 | 793 | 3208 | 382203 | 649 |
| **Huelva** | 101 | -0.04 | 0.63 | 1.63 | 1597 | 203766 | 1482 | 1818 | 209652 | 1332 | 1326 | 217359 | 754 | 1558 | 223455 | 682 |
| **Jaén** | 76 | -0.52 | 0.16 | 1.02 | 1115 | 149634 | 1207 | 1349 | 167991 | 1173 | 1034 | 160620 | 768 | 1283 | 179760 | 701 |
| **Las Palmas** | 263 | -0.17 | 0.68 | 1.49 | 4105 | 520818 | 1383 | 4443 | 556139 | 1147 | 3455 | 542456 | 804 | 3944 | 580661 | 683 |
| **Logroño** | 91 | -0.83 | -0.33 | 0.03 | 1644 | 179285 | 1030 | 1702 | 206196 | 982 | 1357 | 193654 | 618 | 1476 | 218136 | 464 |
| **Lugo** | 69 | -0.68 | -0.11 | 0.24 | 402 | 40753 | 1119 | 1303 | 130626 | 995 | 343 | 45867 | 604 | 1268 | 148948 | 545 |
| **Madrid** | 2358 | -0.95 | -0.30 | 0.32 | 39953 | 4032564 | 1180 | 39811 | 4445163 | 1039 | 36974 | 4591632 | 621 | 40485 | 5003682 | 565 |
| **Málaga** | 422 | 0.06 | 0.78 | 1.37 | 6218 | 787353 | 1357 | 6729 | 789480 | 1212 | 5767 | 850308 | 784 | 6503 | 850323 | 699 |
| **Murcia** | 295 | -0.55 | 0.16 | 0.75 | 3763 | 509007 | 1206 | 4468 | 590310 | 1178 | 3462 | 538113 | 713 | 4126 | 606135 | 693 |
| **Ourense** | 79 | -0.49 | 0.01 | 0.36 | 476 | 50698 | 1082 | 1584 | 150714 | 975 | 445 | 57267 | 642 | 1523 | 172957 | 543 |
| **Oviedo** | 173 | -0.78 | -0.33 | 0.06 | 2776 | 279704 | 1215 | 3011 | 299805 | 1038 | 2493 | 319643 | 627 | 2980 | 343859 | 546 |
| **Pamplona** | 122 | -0.93 | -0.38 | -0.06 | 2324 | 235811 | 1154 | 2462 | 256525 | 995 | 2102 | 260182 | 593 | 2356 | 281458 | 506 |
| **Pontevedra** | 57 | -0.77 | 0.03 | 0.94 | 306 | 34925 | 1290 | 909 | 114008 | 987 | 293 | 38950 | 693 | 919 | 125662 | 541 |
| **San Sebastián** | 140 | -0.99 | -0.59 | -0.12 | 2514 | 248643 | 1308 | 2525 | 253803 | 1002 | 2629 | 282081 | 675 | 2735 | 287826 | 517 |
| **Santa Cruz** | 157 | -0.46 | 0.55 | 1.06 | 2435 | 299467 | 1277 | 2603 | 319962 | 1081 | 2004 | 325477 | 687 | 2334 | 343554 | 611 |
| **Santiago** | 73 | -1.10 | -0.75 | -0.17 | 372 | 43972 | 1237 | 1132 | 130870 | 993 | 331 | 49612 | 603 | 1148 | 149219 | 533 |
| **Sevilla** | 510 | -0.53 | 0.44 | 1.41 | 8134 | 1000752 | 1357 | 8752 | 1009047 | 1207 | 8011 | 1092348 | 783 | 8565 | 1103868 | 665 |
| **Valencia** | 553 | -0.68 | -0.09 | 0.37 | 10910 | 1062004 | 1352 | 10408 | 1154413 | 1079 | 9742 | 1172665 | 737 | 10495 | 1245094 | 614 |
| **Vigo** | 236 | -0.37 | 0.13 | 0.58 | 1011 | 133705 | 1136 | 3618 | 420544 | 1041 | 957 | 148086 | 626 | 3441 | 461208 | 561 |
| **Vitoria** | 168 | -0.56 | -0.10 | 0.29 | 2446 | 315657 | 1217 | 2776 | 338946 | 975 | 2076 | 327045 | 656 | 2401 | 349380 | 504 |
| **Zaragoza** | 462 | -0.70 | -0.13 | 0.33 | 8115 | 888702 | 1068 | 8985 | 926805 | 1115 | 7272 | 956013 | 590 | 8503 | 989592 | 608 |

ASMR: Mortality rate per 100,000 inhabitants, standardised for age by the direct method for the 2001 Spanish population.

P_25_ = 25 Percentile. P_50_ = 50 Percentile. P_75_ = 75 Percentile.
