# Supplementary material for: Trends in socioeconomic inequalities in mortality in small areas of 33 Spanish cities
Source: BMC Public Health. 2016 Jul 29;16:663. doi: 10.1186/s12889-016-3190-y (PMC4966571; doi:10.1186/s12889-016-3190-y)

Atlas of mortality in 33 Spanish cities (periods 1996-1998 and 2005-2007)

Figure 1. Distribution of deprivation index (a) and of the smoothed Standardised Mortality Ratios (sSMR) (b-e) for all-cause mortality, by period (1996-1998 and 2005-2007) and by sex in the city of Alicante. Green areas represent less socioeconomic deprivation and lower sSMR values. Brown areas represent greater socioeconomic deprivation and higher sSMR values.

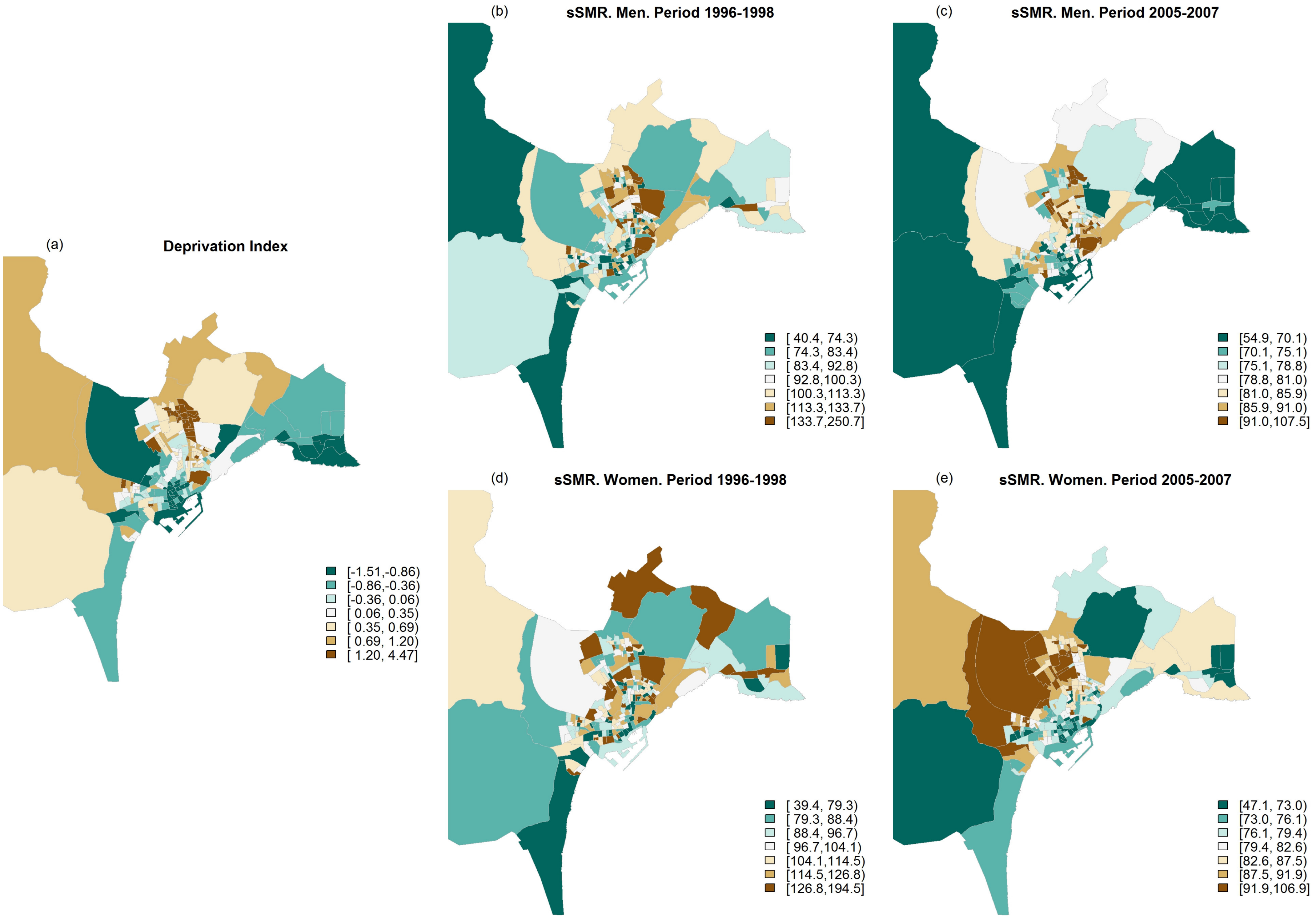

Figure 2. Distribution of deprivation index (a) and of the smoothed Standardised Mortality Ratios (sSMR) (b-e) for all-cause mortality, by period (1996-1998 and 2005-2007) and by sex in the city of Almería. Green areas represent less socioeconomic deprivation and lower sSMR values. Brown areas represent greater socioeconomic deprivation and higher sSMR values.

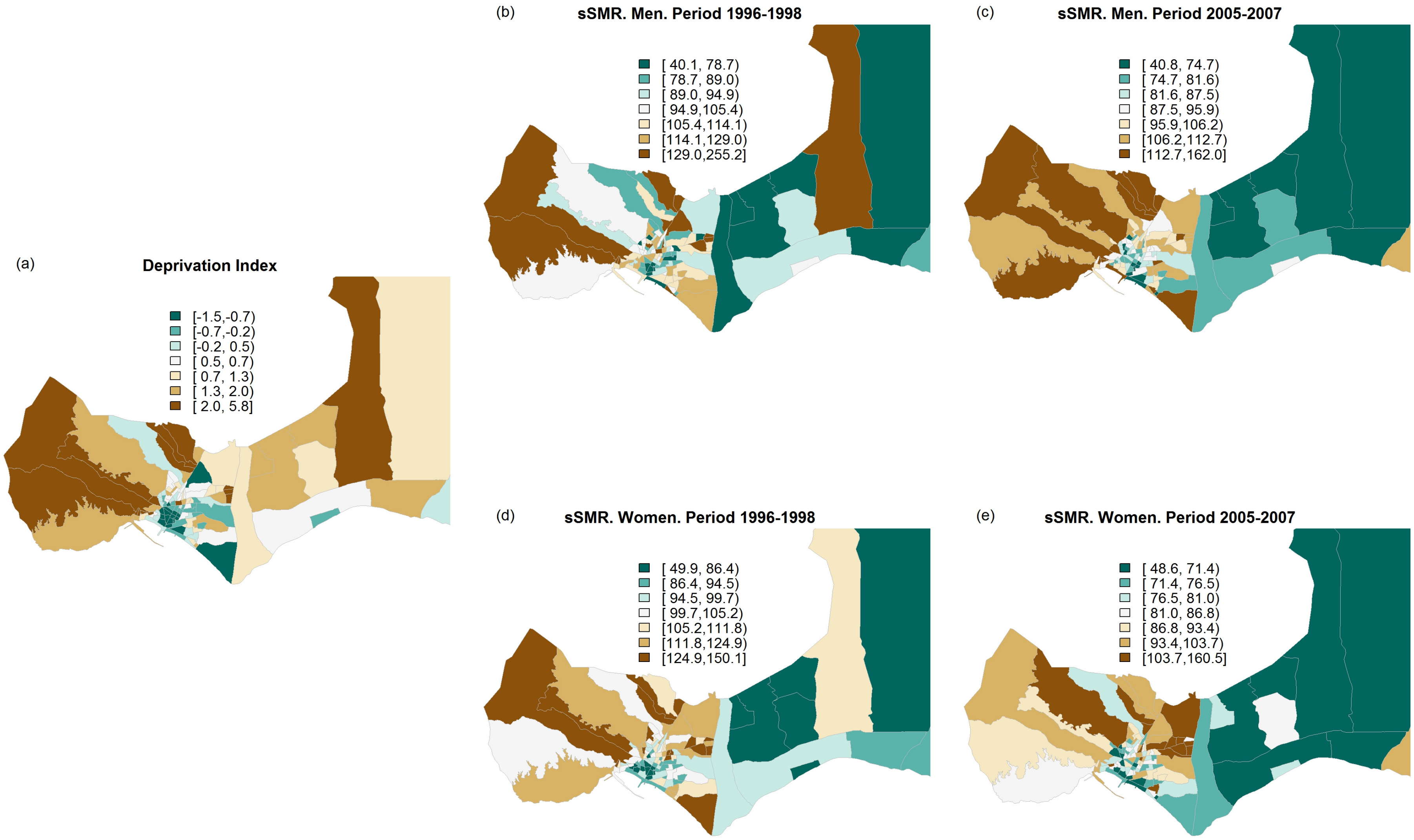

Figure 3. Distribution of deprivation index (a) and of the smoothed Standardised Mortality Ratios (sSMR) (b-e) for all-cause mortality, by period (1996-1998 and 2005-2007) and by sex in the city of Avilés. Green areas represent less socioeconomic deprivation and lower sSMR values. Brown areas represent greater socioeconomic deprivation and higher sSMR values.

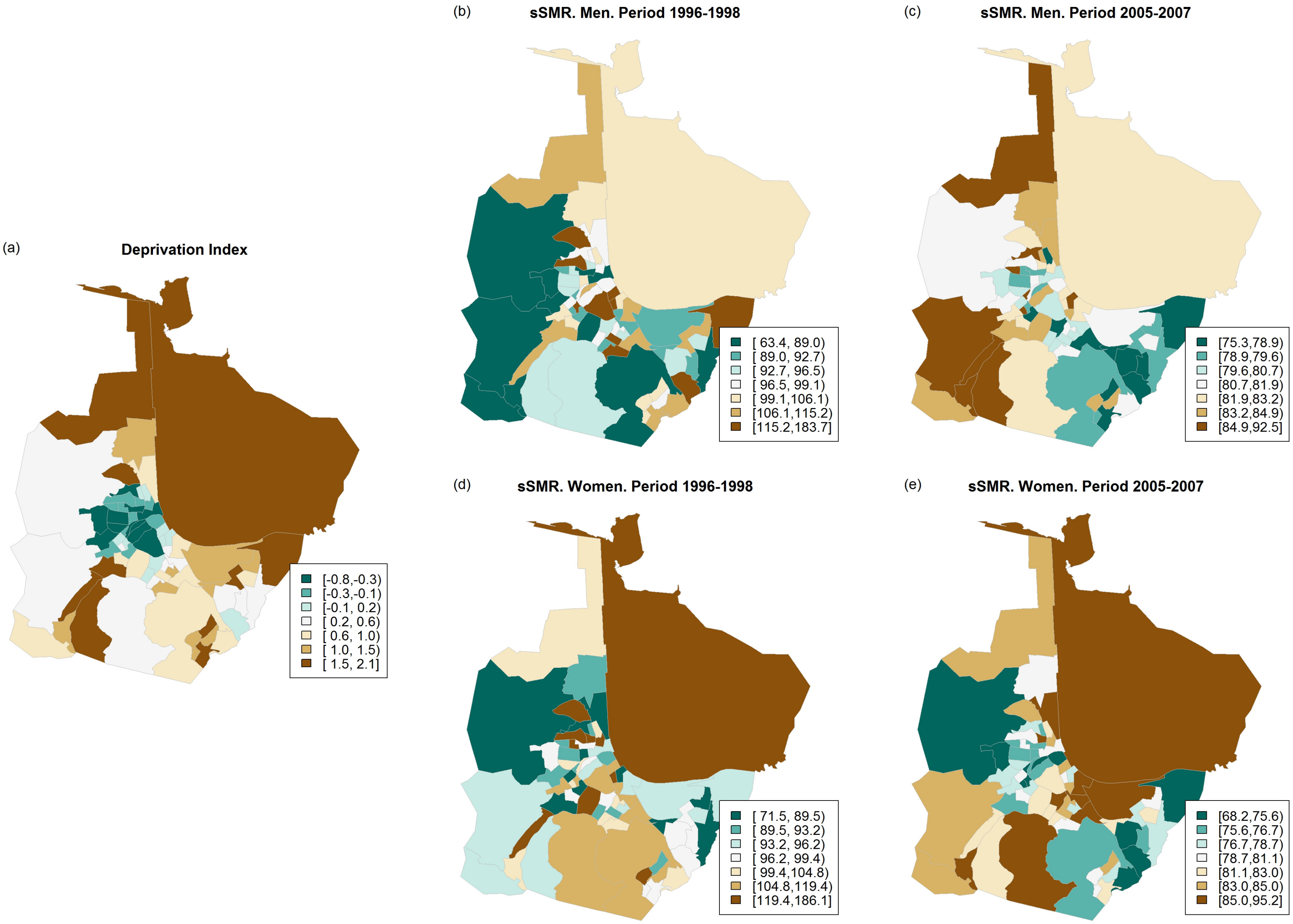

Figure 4. Distribution of deprivation index (a) and of the smoothed Standardised Mortality Ratios (sSMR) (b-e) for all-cause mortality, by period (1996-1998 and 2005-2007) and by sex in the city of Barcelona. Green areas represent less socioeconomic deprivation and lower sSMR values. Brown areas represent greater socioeconomic deprivation and higher sSMR values.

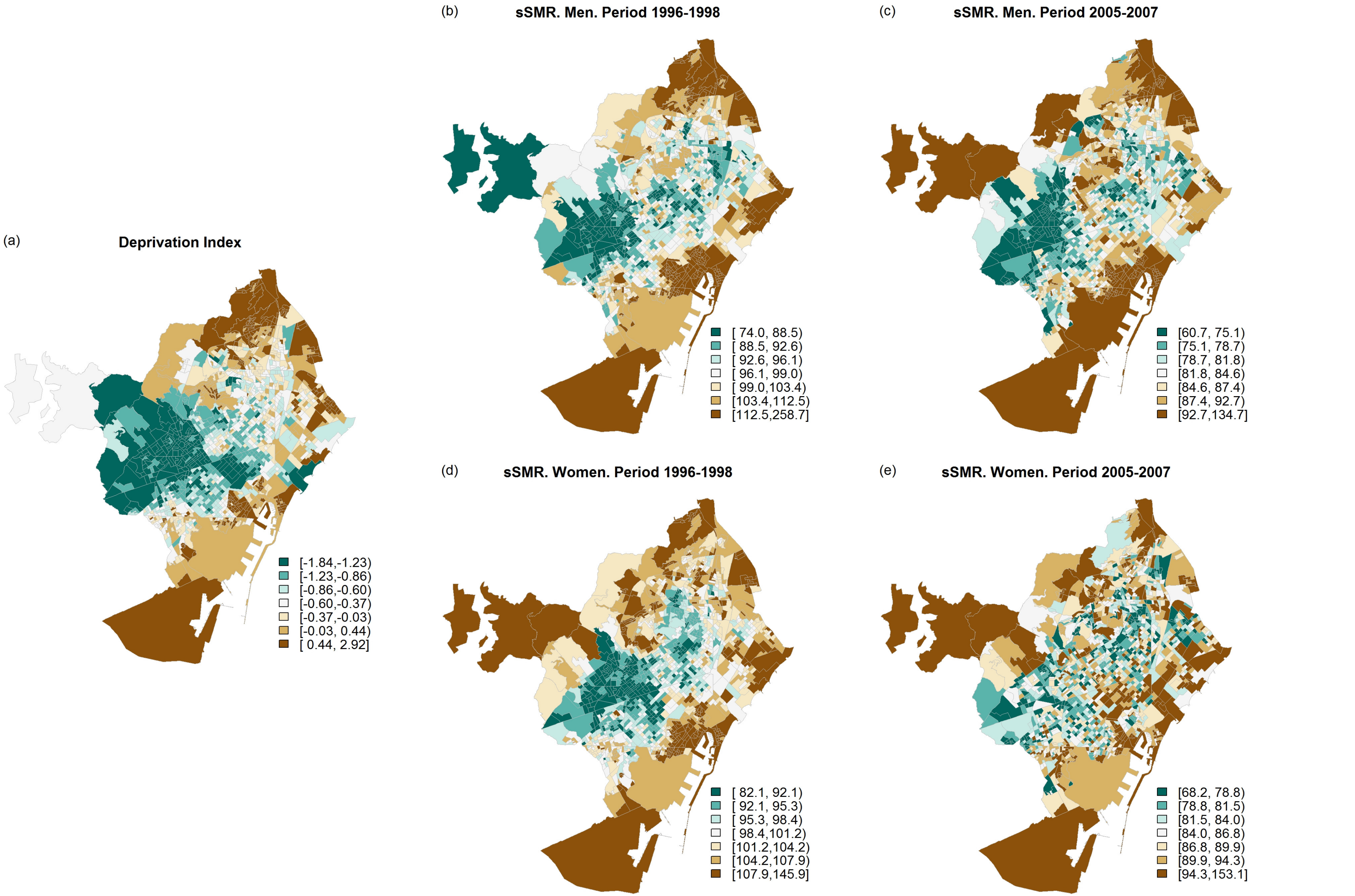

Figure 5. Distribution of deprivation index (a) and of the smoothed Standardised Mortality Ratios (sSMR) (b-e) for all-cause mortality, by period (1996-1998 and 2005-2007) and by sex in the city of Bilbao. Green areas represent less socioeconomic deprivation and lower sSMR values. Brown areas represent greater socioeconomic deprivation and higher sSMR values.

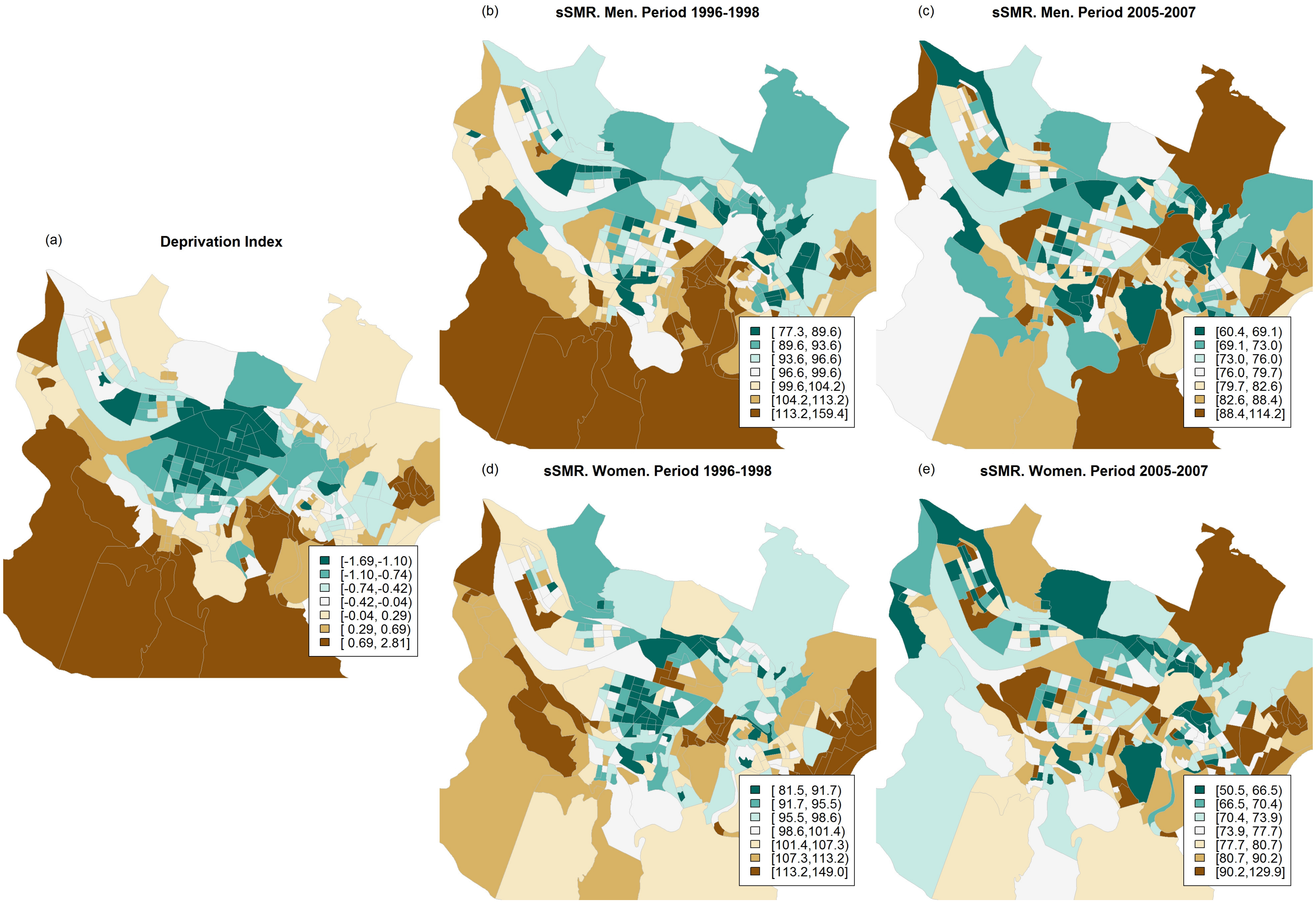

Figure 6. Distribution of deprivation index (a) and of the smoothed Standardised Mortality Ratios (sSMR) (b-e) for all-cause mortality, by period (1996-1998 and 2005-2007) and by sex in the city of Cádiz. Green areas represent less socioeconomic deprivation and lower sSMR values. Brown areas represent greater socioeconomic deprivation and higher sSMR values.

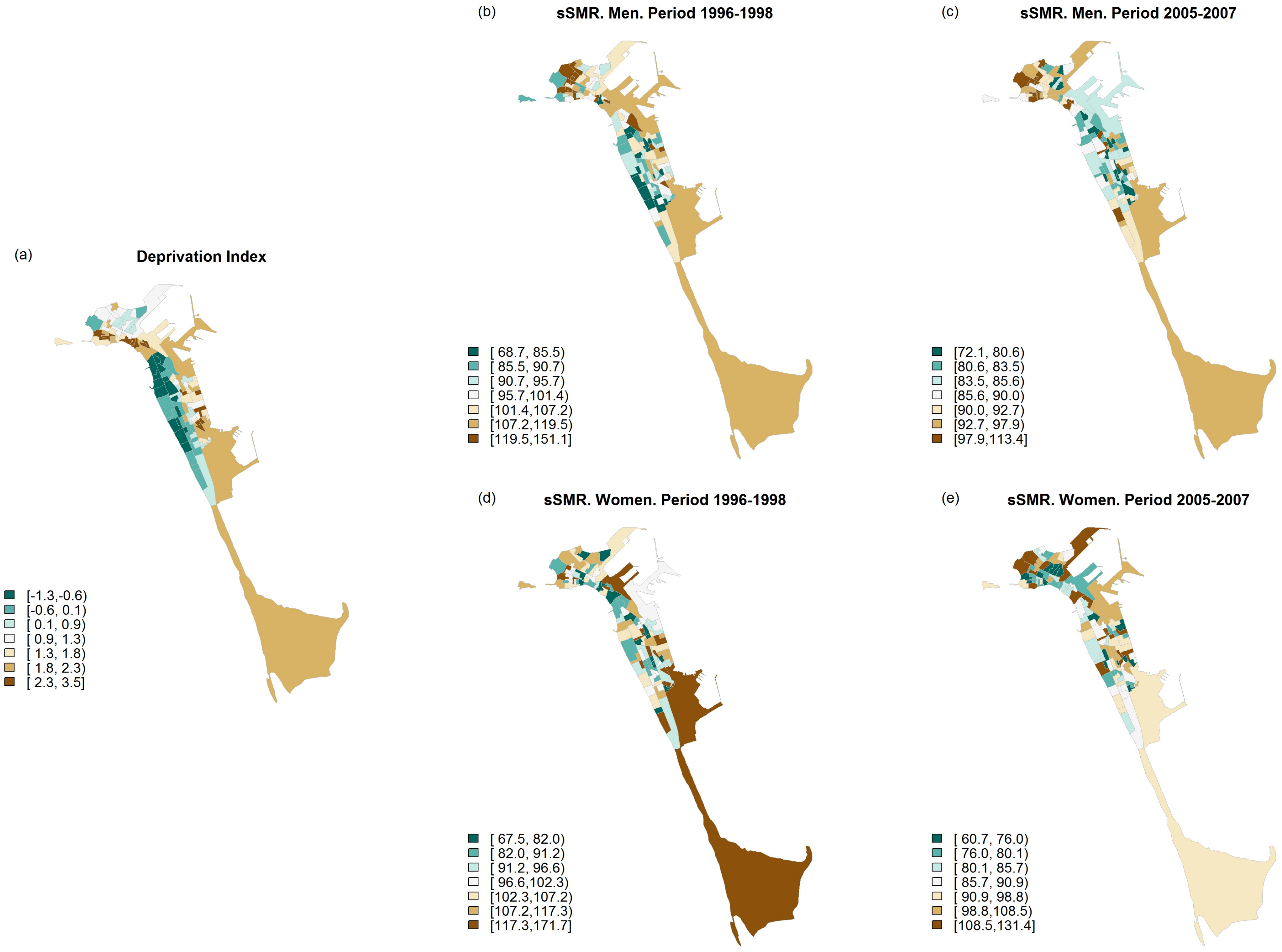

Figure 7. Distribution of deprivation index (a) and of the smoothed Standardised Mortality Ratios (sSMR) (b-e) for all-cause mortality, by period (1996-1998 and 2005-2007) and by sex in the city of Cartagena-La Unión. Green areas represent less socioeconomic deprivation and lower sSMR values. Brown areas represent greater socioeconomic deprivation and higher sSMR values.

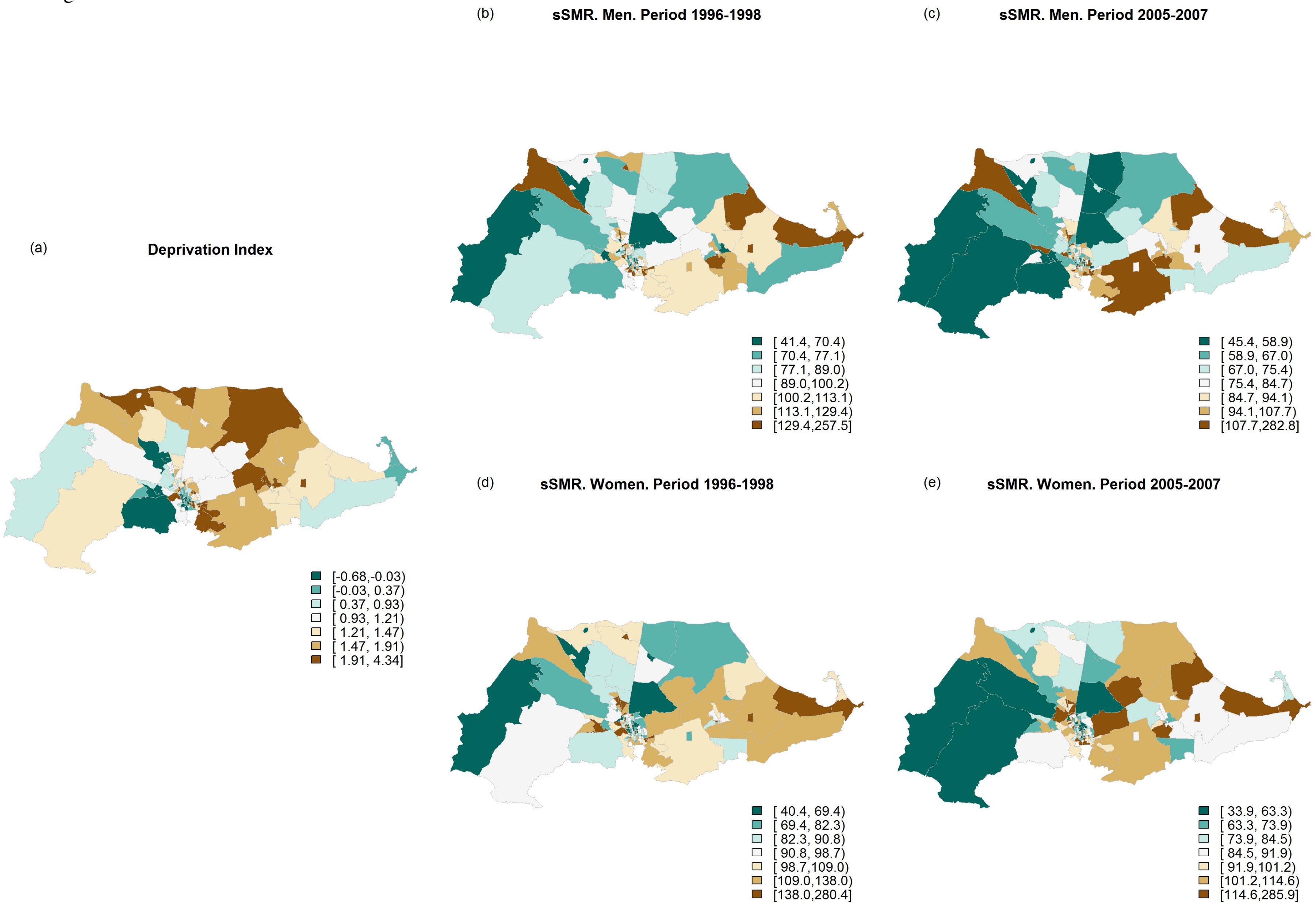

Figure 8. Distribution of deprivation index (a) and of the smoothed Standardised Mortality Ratios (sSMR) (b-e) for all-cause mortality, by period (1996-1998 and 2005-2007) and by sex in the city of Castellón. Green areas represent less socioeconomic deprivation and lower sSMR values. Brown areas represent greater socioeconomic deprivation and higher sSMR values.

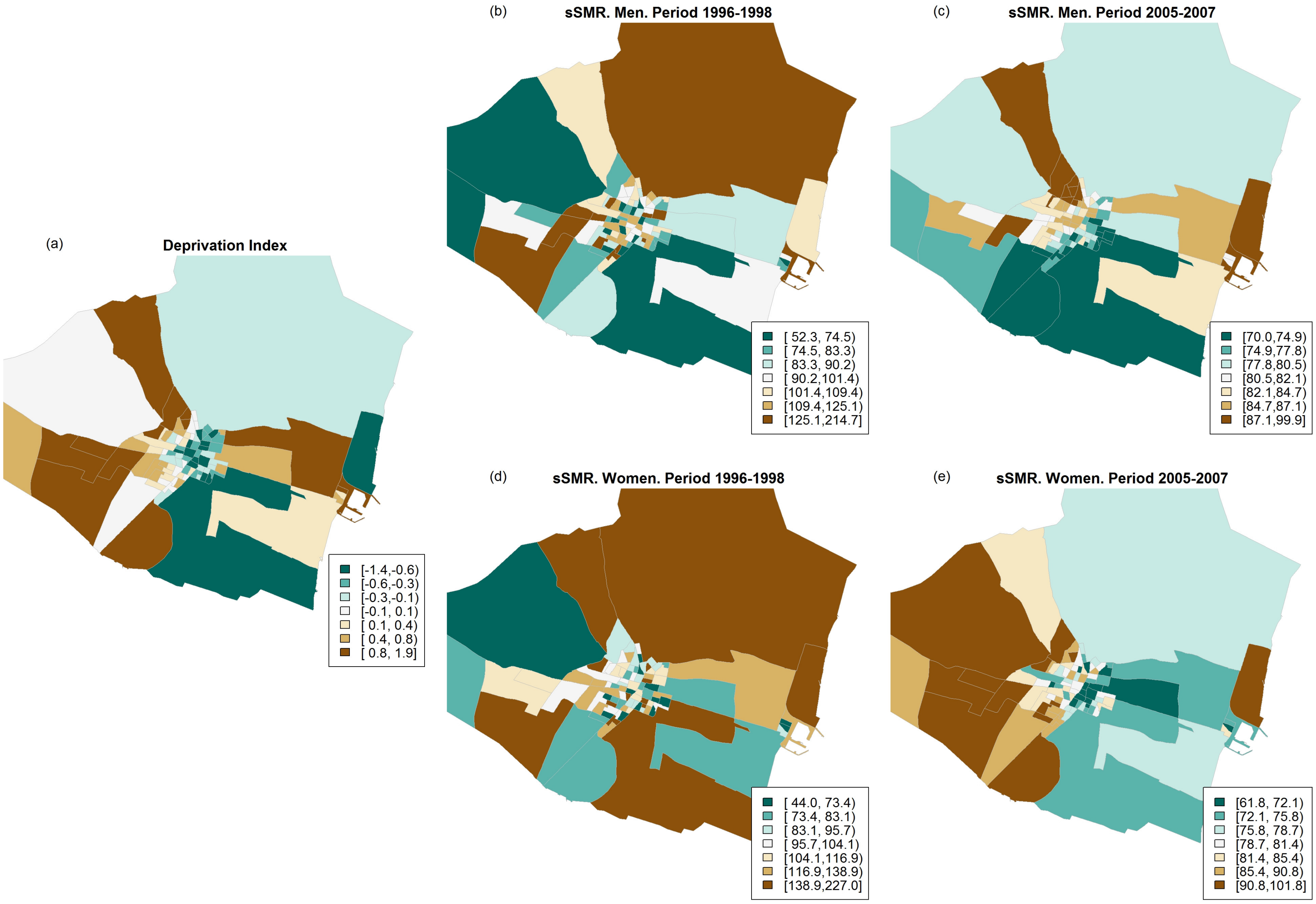

Figure 9. Distribution of deprivation index (a) and of the smoothed Standardised Mortality Ratios (sSMR) (b-e) for all-cause mortality, by period (1996-1998 and 2005-2007) and by sex in the city of Córdoba. Green areas represent less socioeconomic deprivation and lower sSMR values. Brown areas represent greater socioeconomic deprivation and higher sSMR values.

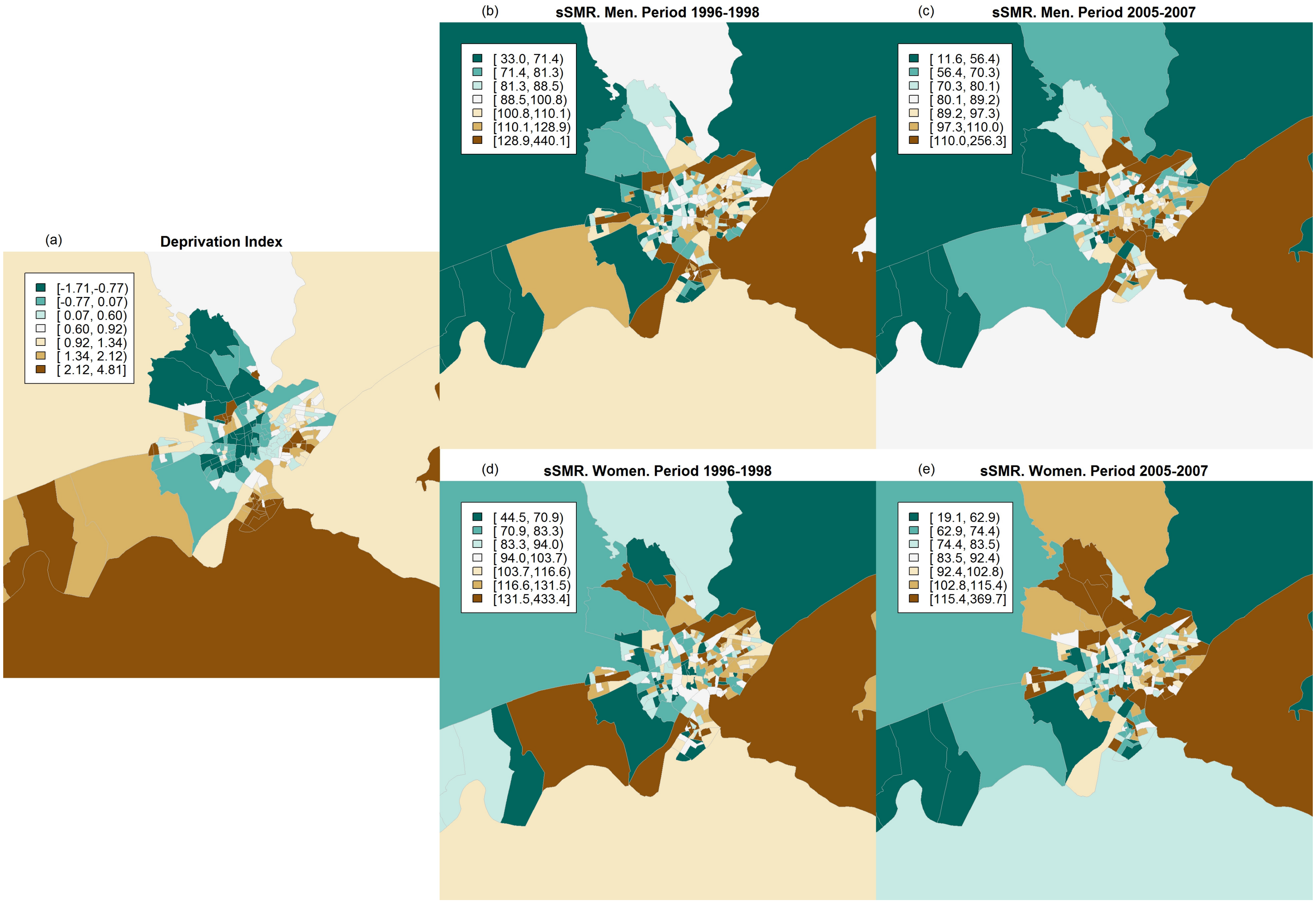

Figure 10. Distribution of deprivation index (a) and of the smoothed Standardised Mortality Ratios (sSMR) (b-e) for all-cause mortality, by period (1996-1998 and 2005-2007) and by sex in the city of Coruña. Green areas represent less socioeconomic deprivation and lower sSMR values. Brown areas represent greater socioeconomic deprivation and higher sSMR values.

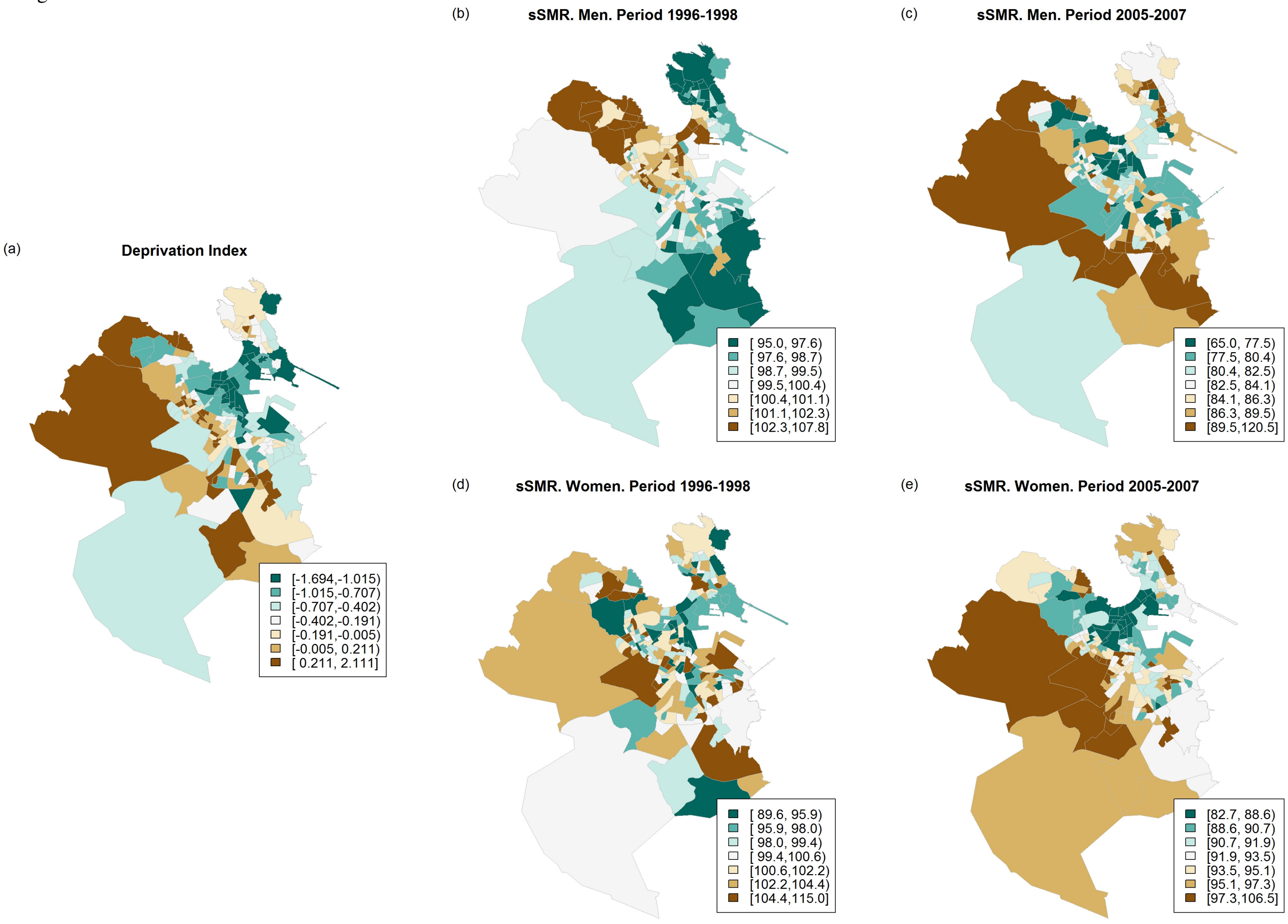

Figure 11. Distribution of deprivation index (a) and of the smoothed Standardised Mortality Ratios (sSMR) (b-e) for all-cause mortality, by period (1996-1998 and 2005-2007) and by sex in the city of Ferrol. Green areas represent less socioeconomic deprivation and lower sSMR values. Brown areas represent greater socioeconomic deprivation and higher sSMR values.

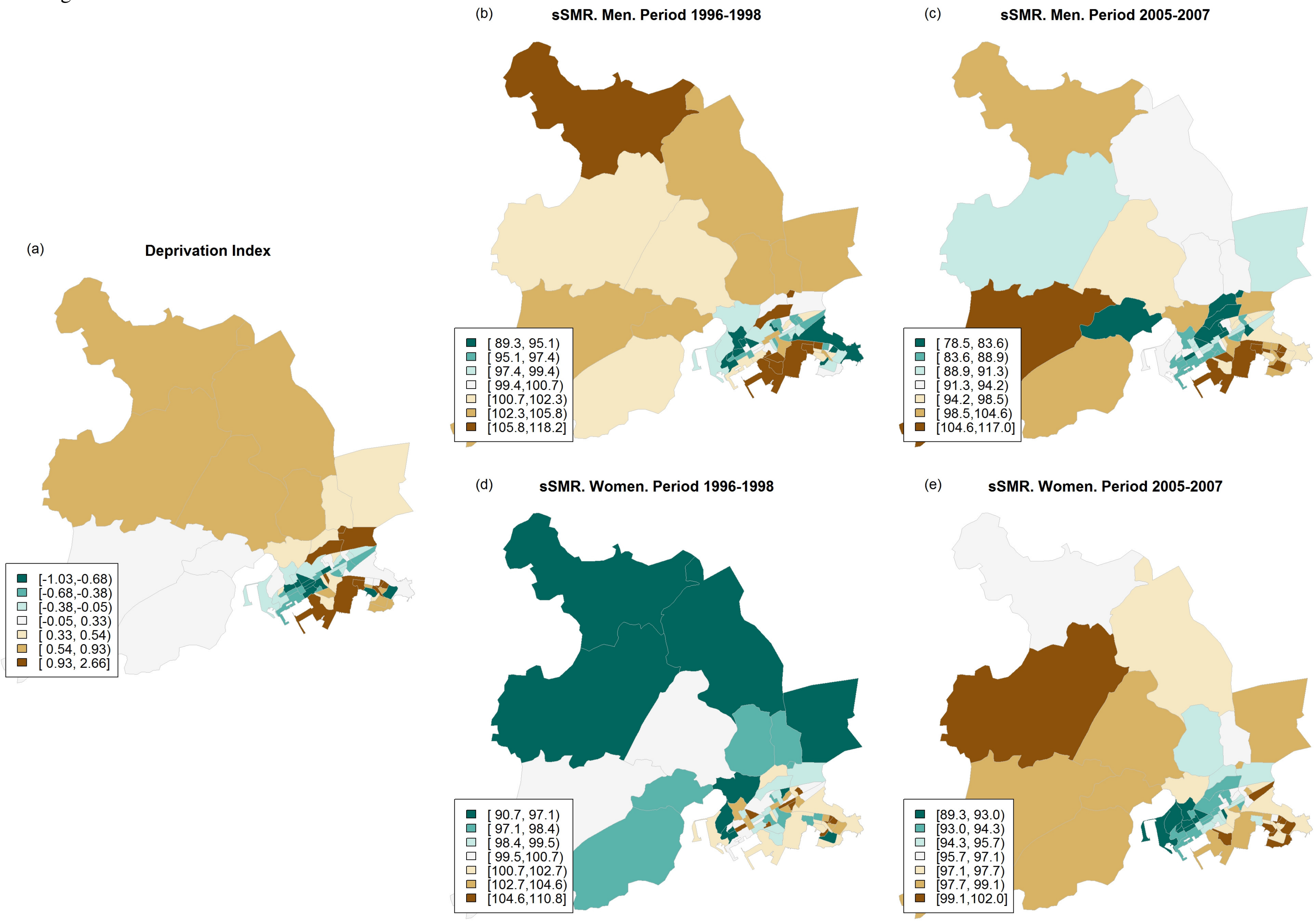

Figure 12. Distribution of deprivation index (a) and of the smoothed Standardised Mortality Ratios (sSMR) (b-e) for all-cause mortality, by period (1996-1998 and 2005-2007) and by sex in the city of Gijón. Green areas represent less socioeconomic deprivation and lower sSMR values. Brown areas represent greater socioeconomic deprivation and higher sSMR values.

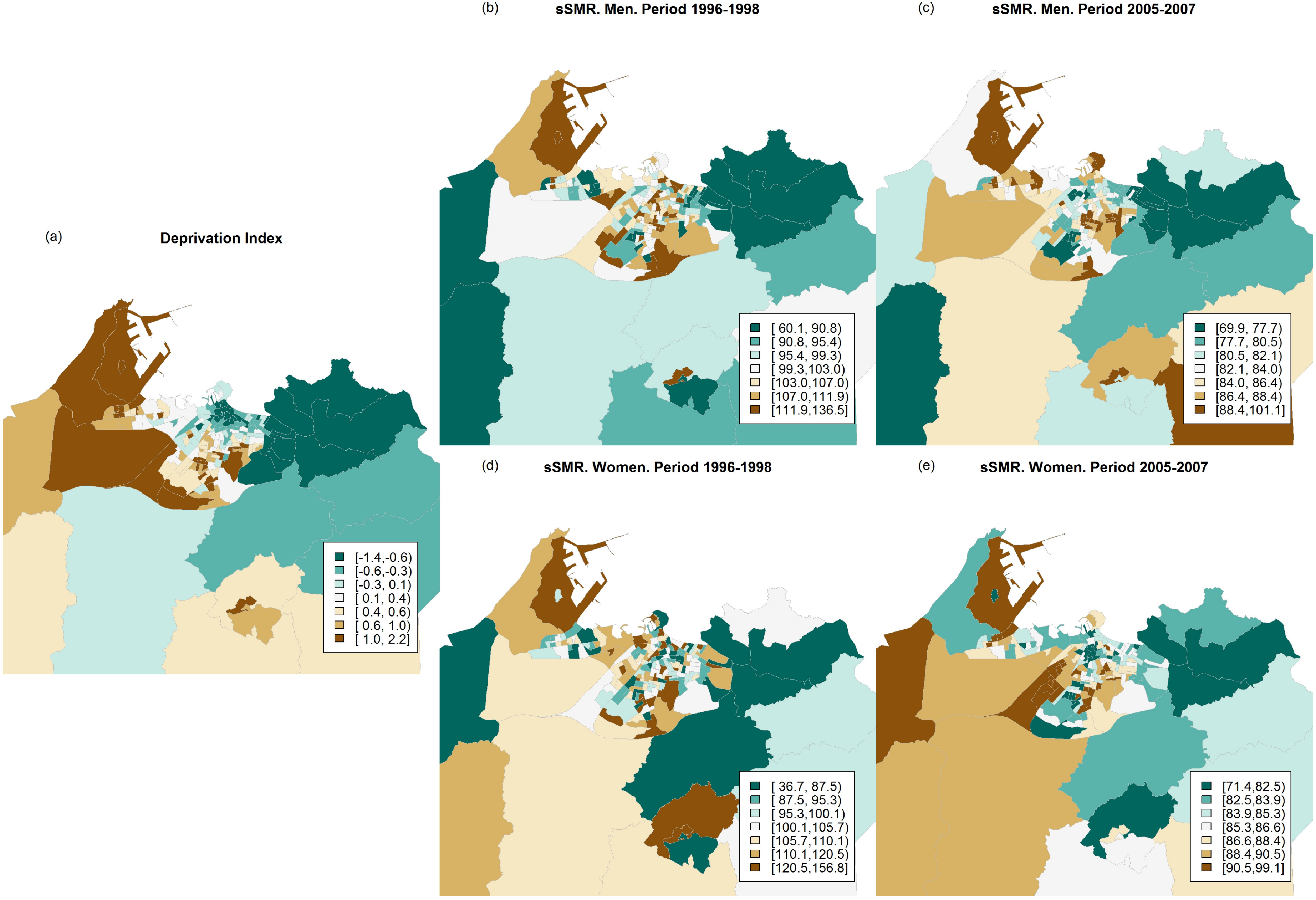

Figure 13. Distribution of deprivation index (a) and of the smoothed Standardised Mortality Ratios (sSMR) (b-e) for all-cause mortality, by period (1996-1998 and 2005-2007) and by sex in the city of Granada. Green areas represent less socioeconomic deprivation and lower sSMR values. Brown areas represent greater socioeconomic deprivation and higher sSMR values.

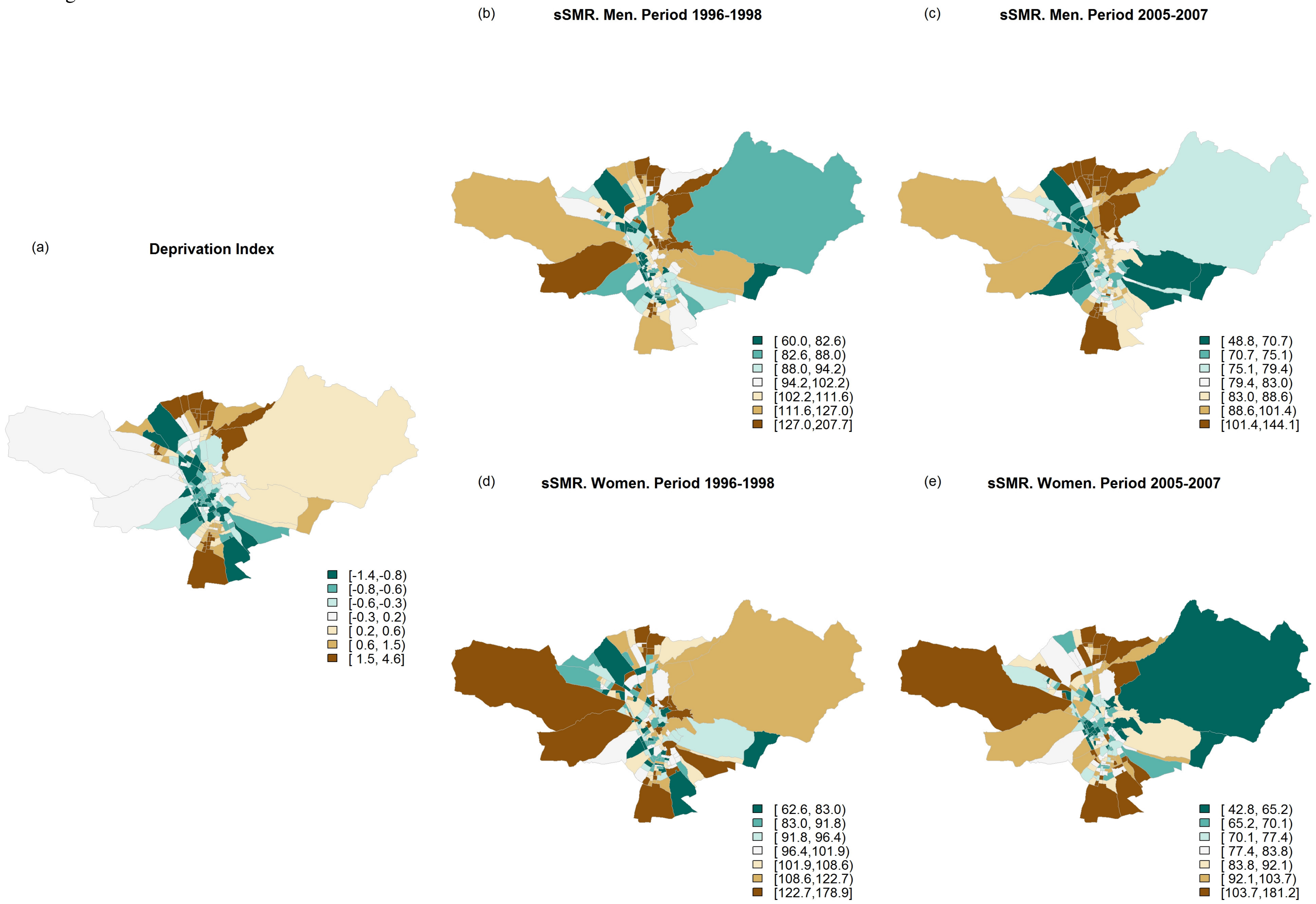

Figure 14. Distribution of deprivation index (a) and of the smoothed Standardised Mortality Ratios (sSMR) (b-e) for all-cause mortality, by period (1996-1998 and 2005-2007) and by sex in the city of Huelva. Green areas represent less socioeconomic deprivation and lower sSMR values. Brown areas represent greater socioeconomic deprivation and higher sSMR values.

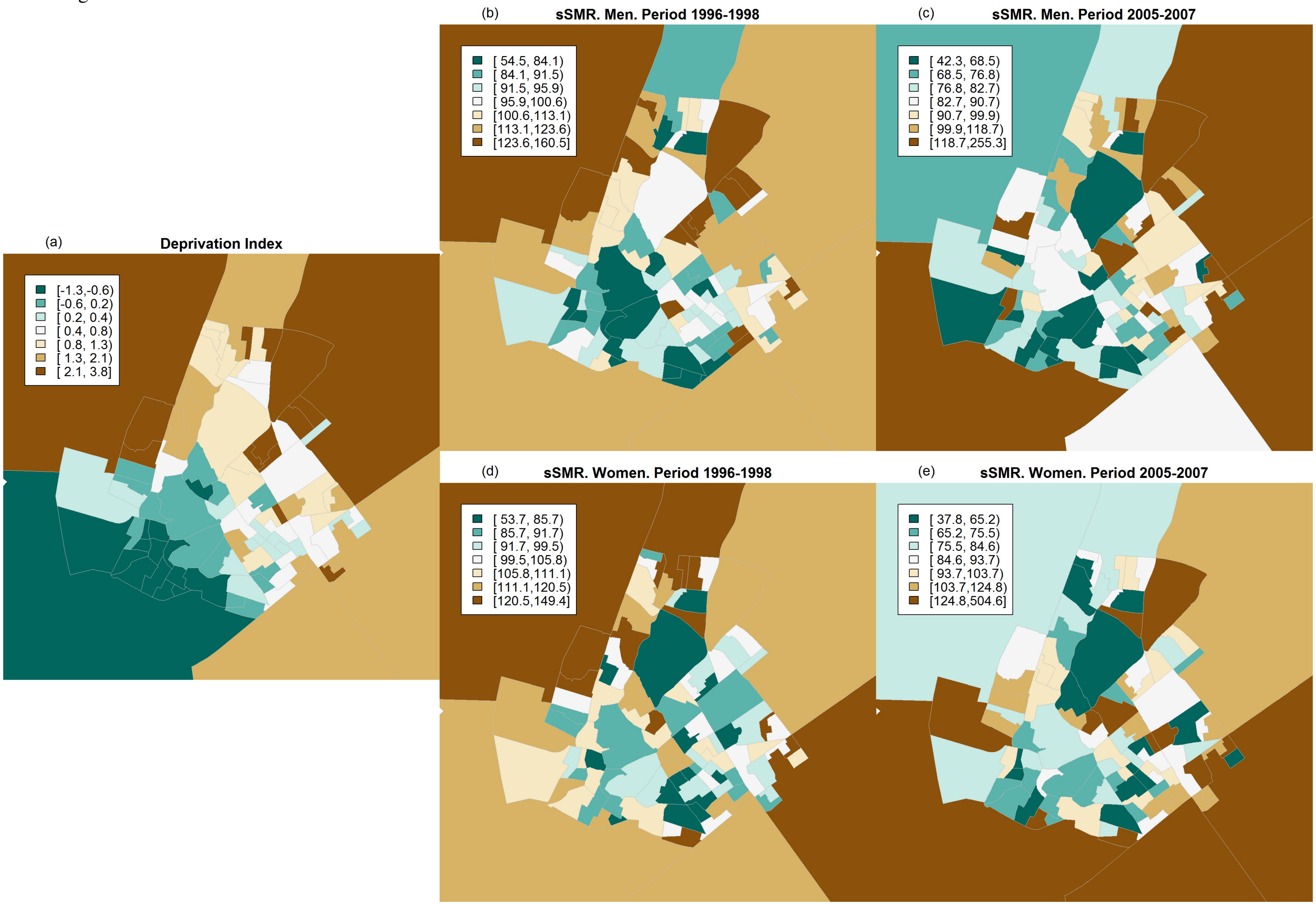

Figure 15. Distribution of deprivation index (a) and of the smoothed Standardised Mortality Ratios (sSMR) (b-e) for all-cause mortality, by period (1996-1998 and 2005-2007) and by sex in the city of Jaén. Green areas represent less socioeconomic deprivation and lower sSMR values. Brown areas represent greater socioeconomic deprivation and higher sSMR values.

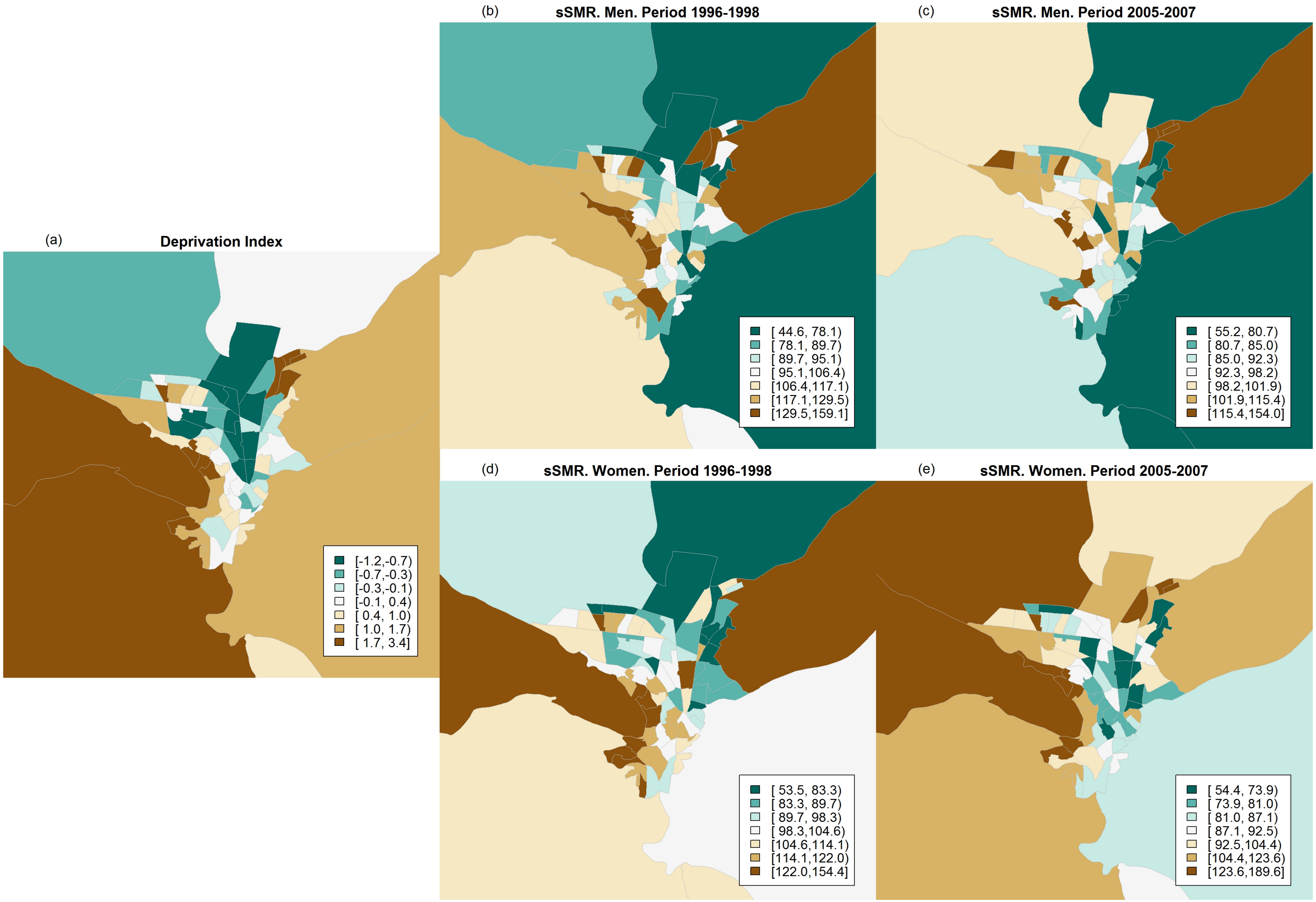

Figure 16. Distribution of deprivation index (a) and of the smoothed Standardised Mortality Ratios (sSMR) (b-e) for all-cause mortality, by period (1996-1998 and 2005-2007) and by sex in the city of Las Palmas. Green areas represent less socioeconomic deprivation and lower sSMR values. Brown areas represent greater socioeconomic deprivation and higher sSMR values.

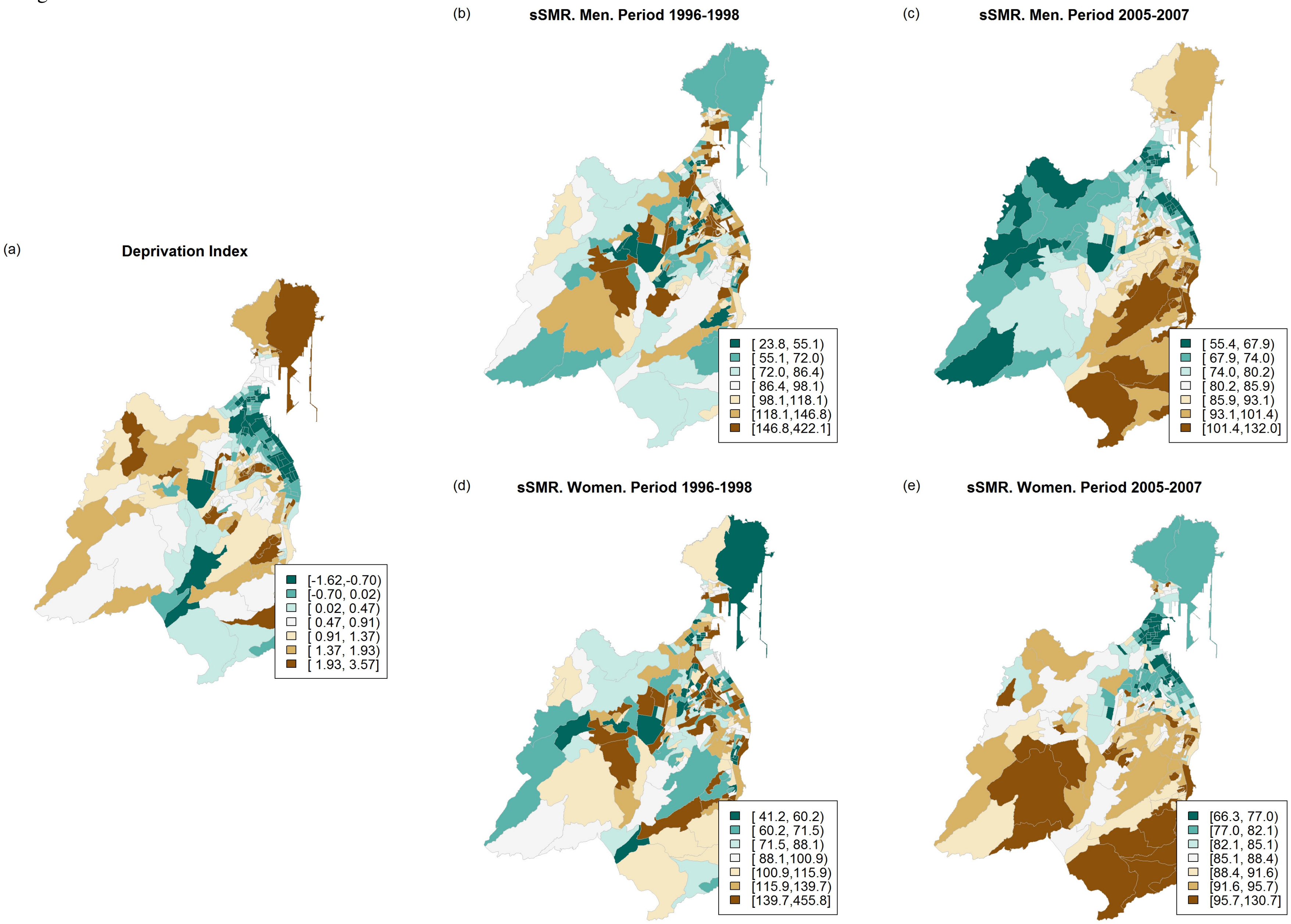

Figure 17. Distribution of deprivation index (a) and of the smoothed Standardised Mortality Ratios (sSMR) (b-e) for all-cause mortality, by period (1996-1998 and 2005-2007) and by sex in the city of Logroño. Green areas represent less socioeconomic deprivation and lower sSMR values. Brown areas represent greater socioeconomic deprivation and higher sSMR values.

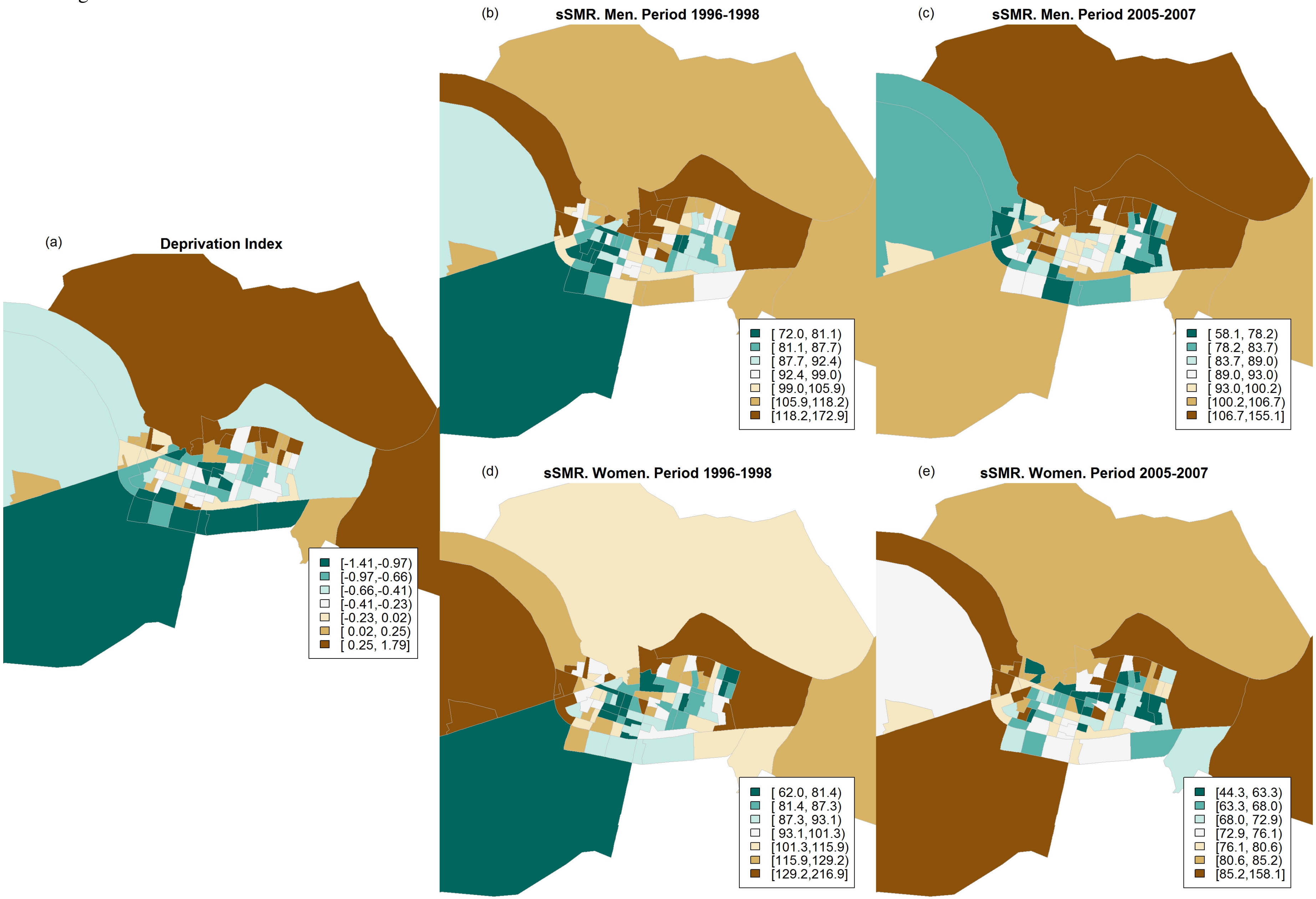

Figure 18. Distribution of deprivation index (a) and of the smoothed Standardised Mortality Ratios (sSMR) (b-e) for all-cause mortality, by period (1996-1998 and 2005-2007) and by sex in the city of Lugo. Green areas represent less socioeconomic deprivation and lower sSMR values. Brown areas represent greater socioeconomic deprivation and higher sSMR values.

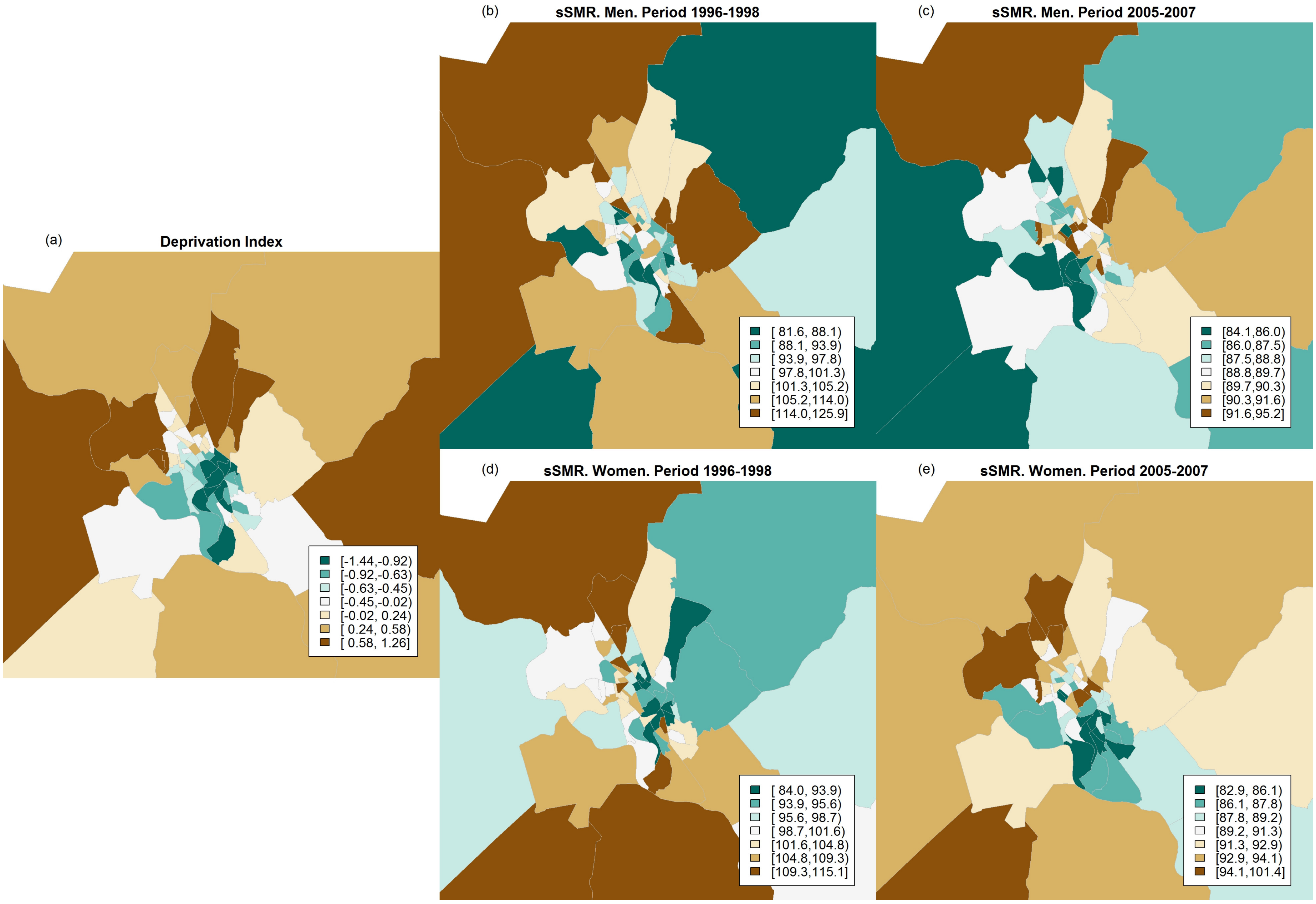

Figure 19. Distribution of deprivation index (a) and of the smoothed Standardised Mortality Ratios (sSMR) (b-e) for all-cause mortality, by period (1996-1998 and 2005-2007) and by sex in the city of Madrid. Green areas represent less socioeconomic deprivation and lower sSMR values. Brown areas represent greater socioeconomic deprivation and higher sSMR values.

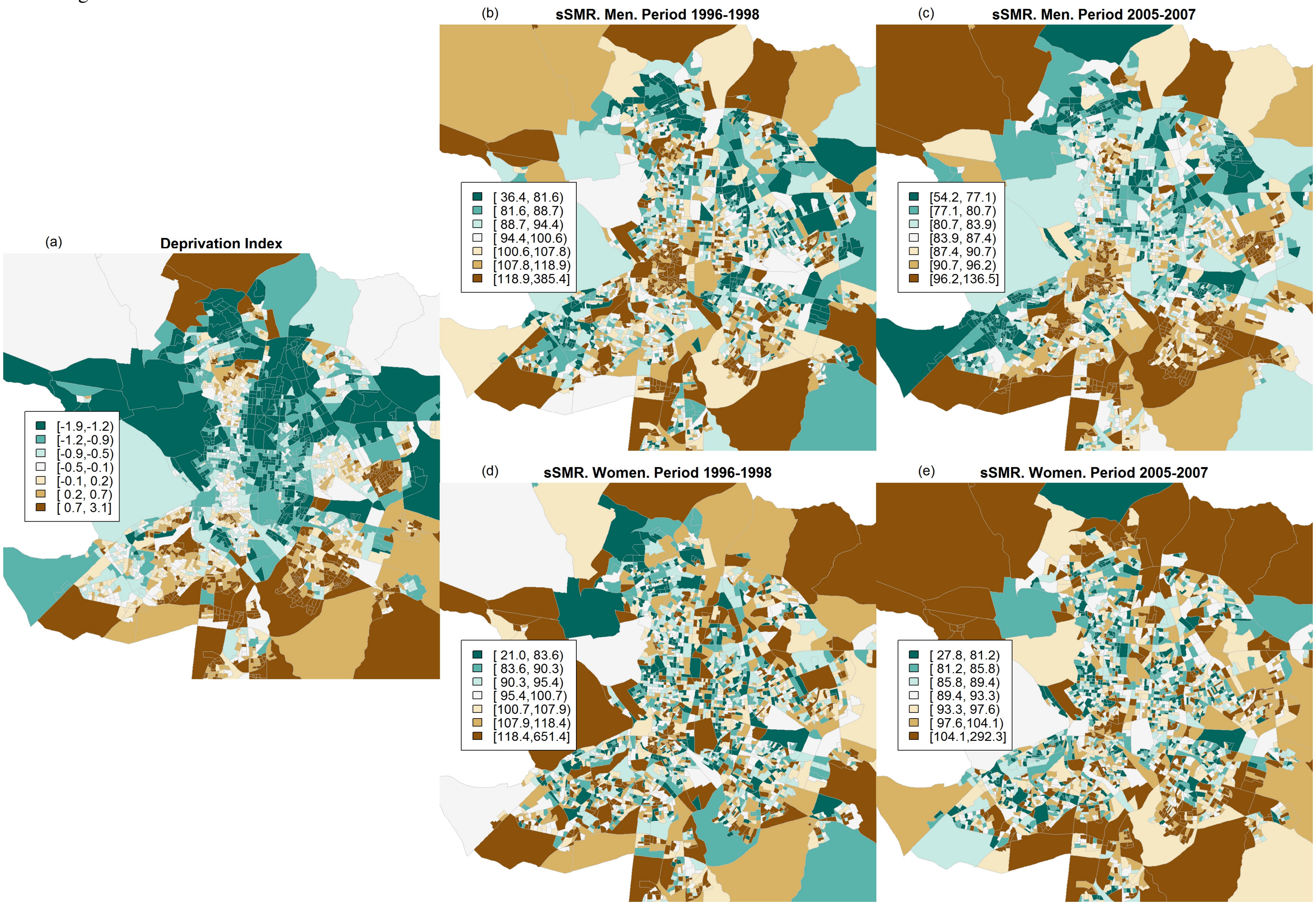

Figure 20. Distribution of deprivation index (a) and of the smoothed Standardised Mortality Ratios (sSMR) (b-e) for all-cause mortality, by period (1996-1998 and 2005-2007) and by sex in the city of Málaga. Green areas represent less socioeconomic deprivation and lower sSMR values. Brown areas represent greater socioeconomic deprivation and higher sSMR values.

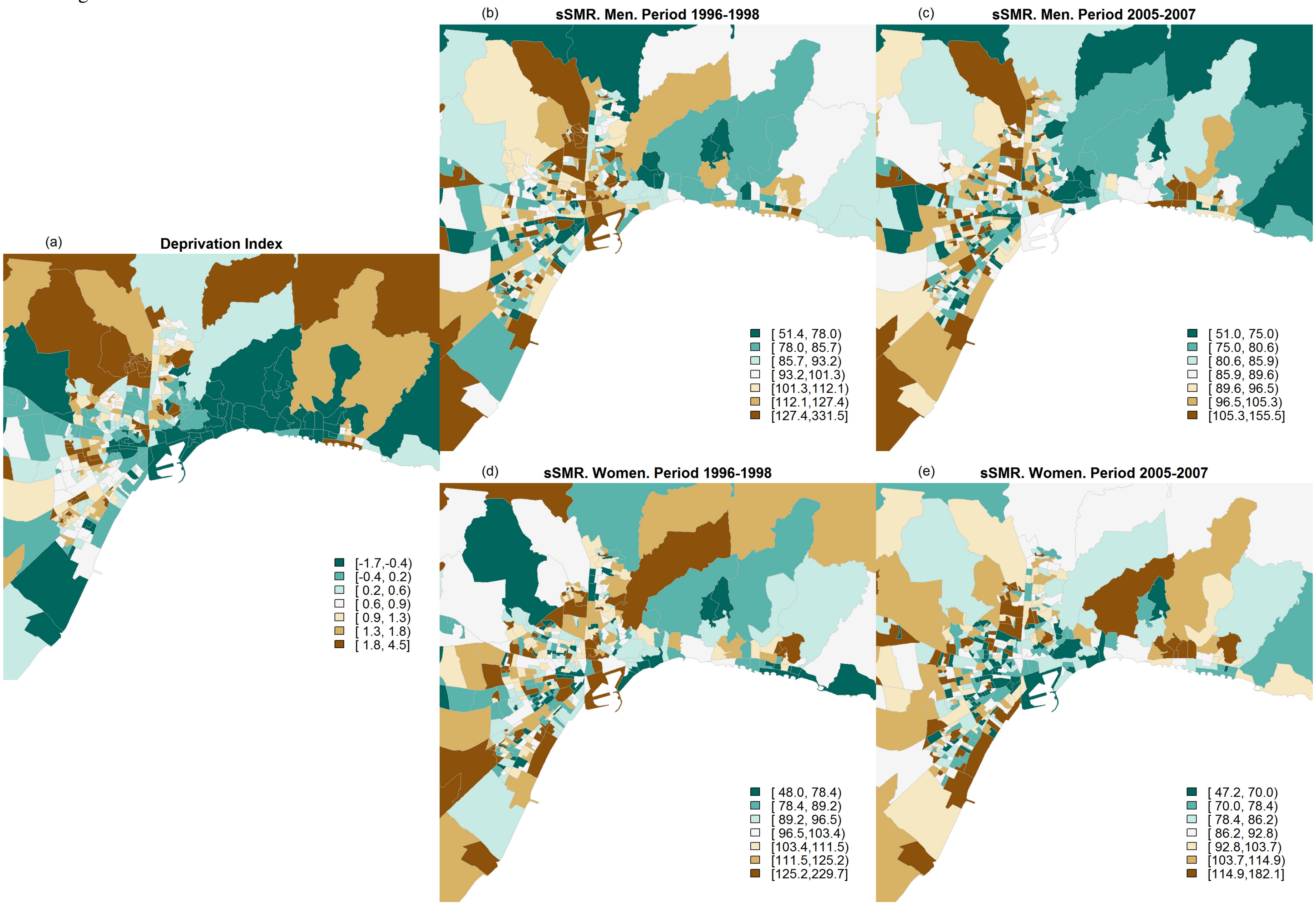

Figure 21. Distribution of deprivation index (a) and of the smoothed Standardised Mortality Ratios (sSMR) (b-e) for all-cause mortality, by period (1996-1998 and 2005-2007) and by sex in the city of Murcia. Green areas represent less socioeconomic deprivation and lower sSMR values. Brown areas represent greater socioeconomic deprivation and higher sSMR values.

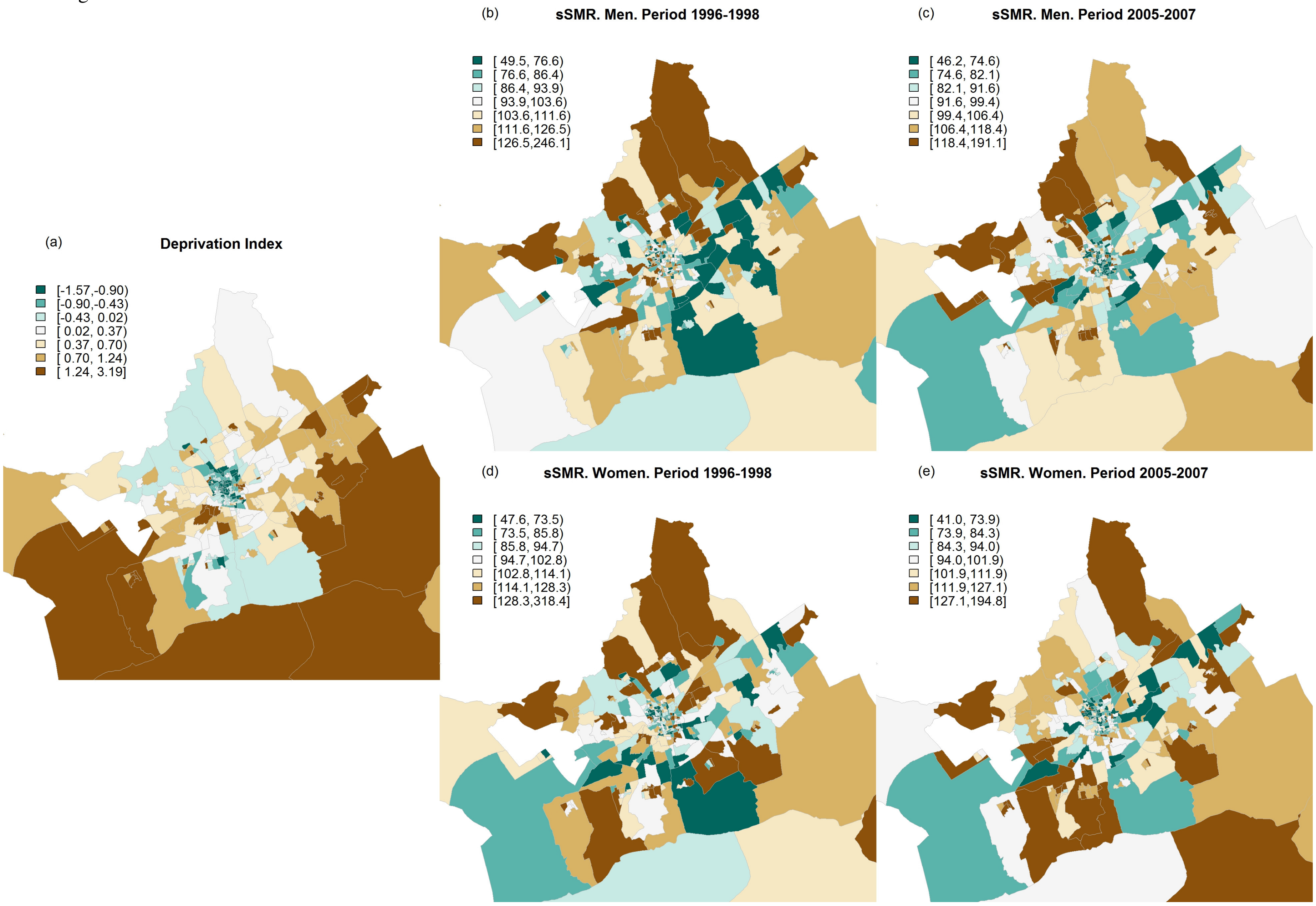

Figure 22. Distribution of deprivation index (a) and of the smoothed Standardised Mortality Ratios (sSMR) (b-e) for all-cause mortality, by period (1996-1998 and 2005-2007) and by sex in the city of Ourense. Green areas represent less socioeconomic deprivation and lower sSMR values. Brown areas represent greater socioeconomic deprivation and higher sSMR values.

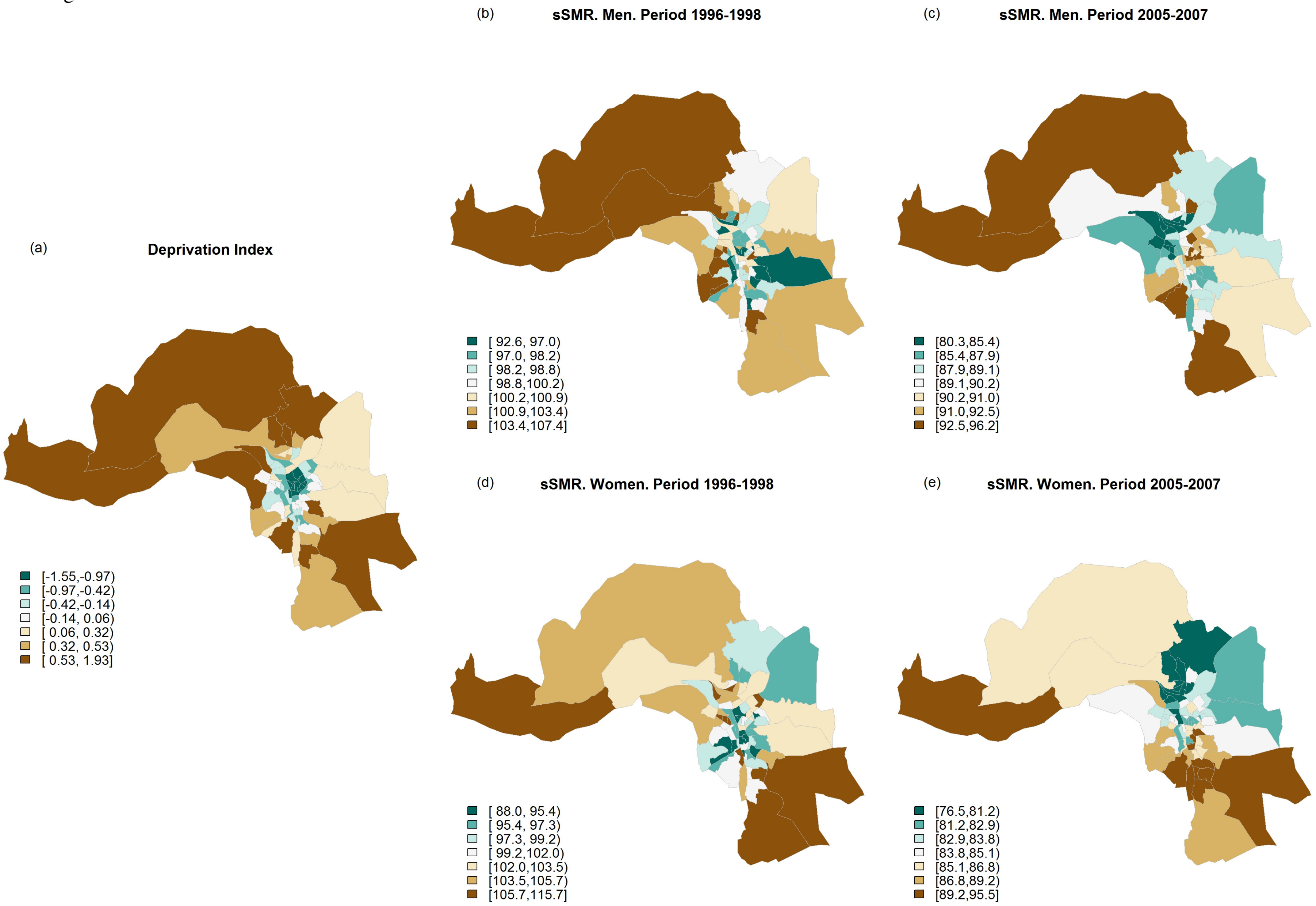

Figure 23. Distribution of deprivation index (a) and of the smoothed Standardised Mortality Ratios (sSMR) (b-e) for all-cause mortality, by period (1996-1998 and 2005-2007) and by sex in the city of Oviedo. Green areas represent less socioeconomic deprivation and lower sSMR values. Brown areas represent greater socioeconomic deprivation and higher sSMR values.

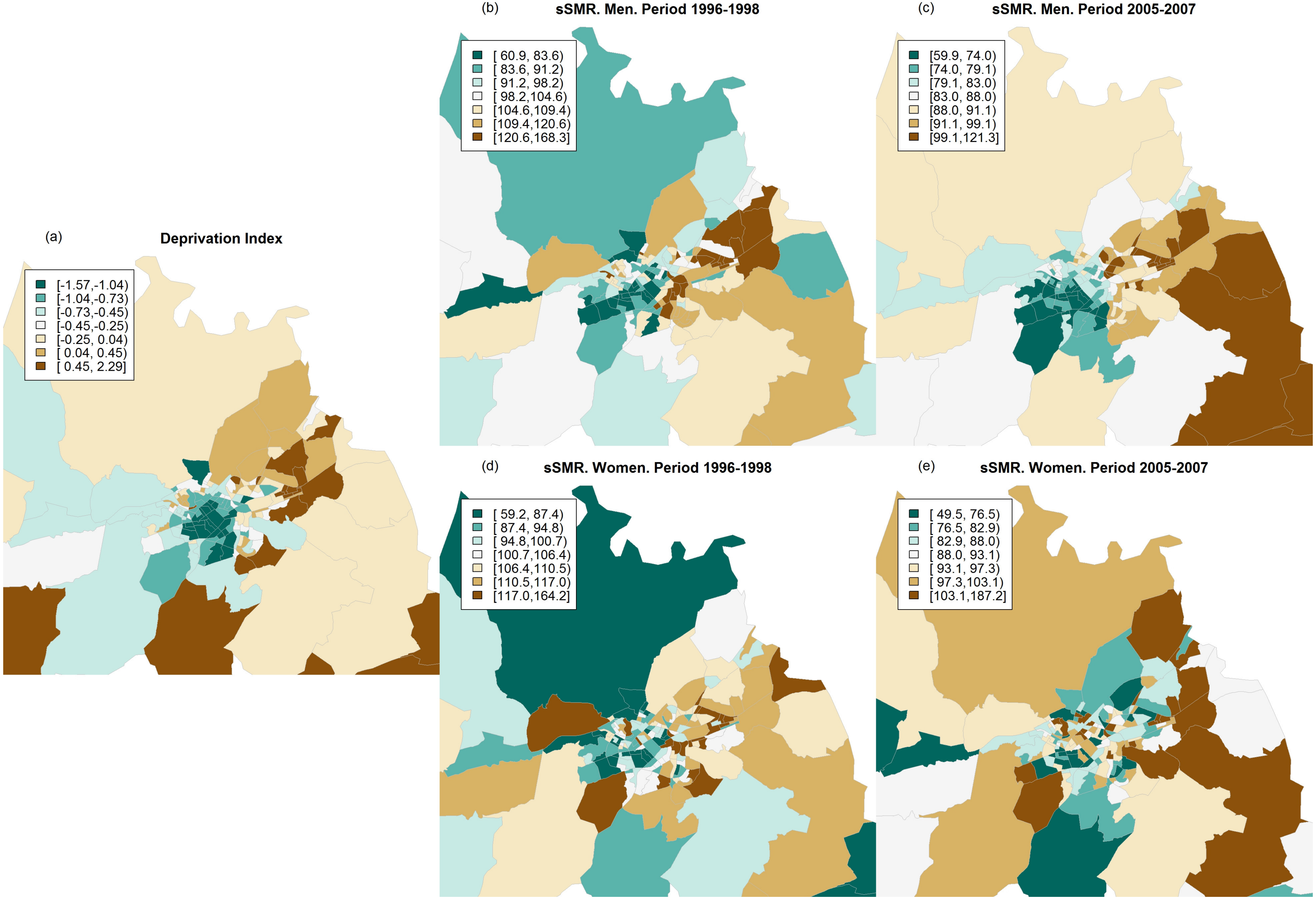

Figure 24. Distribution of deprivation index (a) and of the smoothed Standardised Mortality Ratios (sSMR) (b-e) for all-cause mortality, by period (1996-1998 and 2005-2007) and by sex in the city of Pamplona. Green areas represent less socioeconomic deprivation and lower sSMR values. Brown areas represent greater socioeconomic deprivation and higher sSMR values.

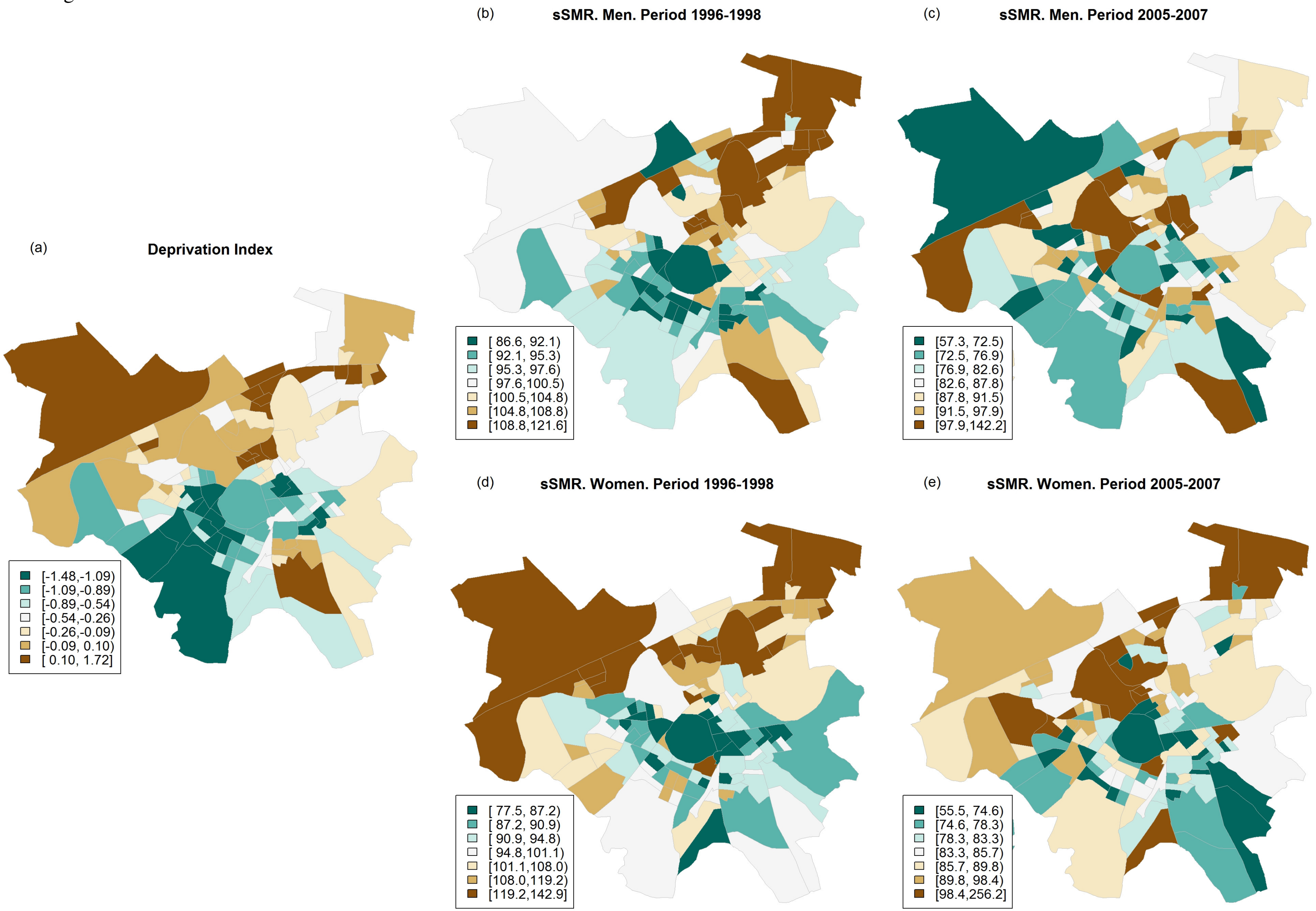

Figure 25. Distribution of deprivation index (a) and of the smoothed Standardised Mortality Ratios (sSMR) (b-e) for all-cause mortality, by period (1996-1998 and 2005-2007) and by sex in the city of Pontevedra. Green areas represent less socioeconomic deprivation and lower sSMR values. Brown areas represent greater socioeconomic deprivation and higher sSMR values.

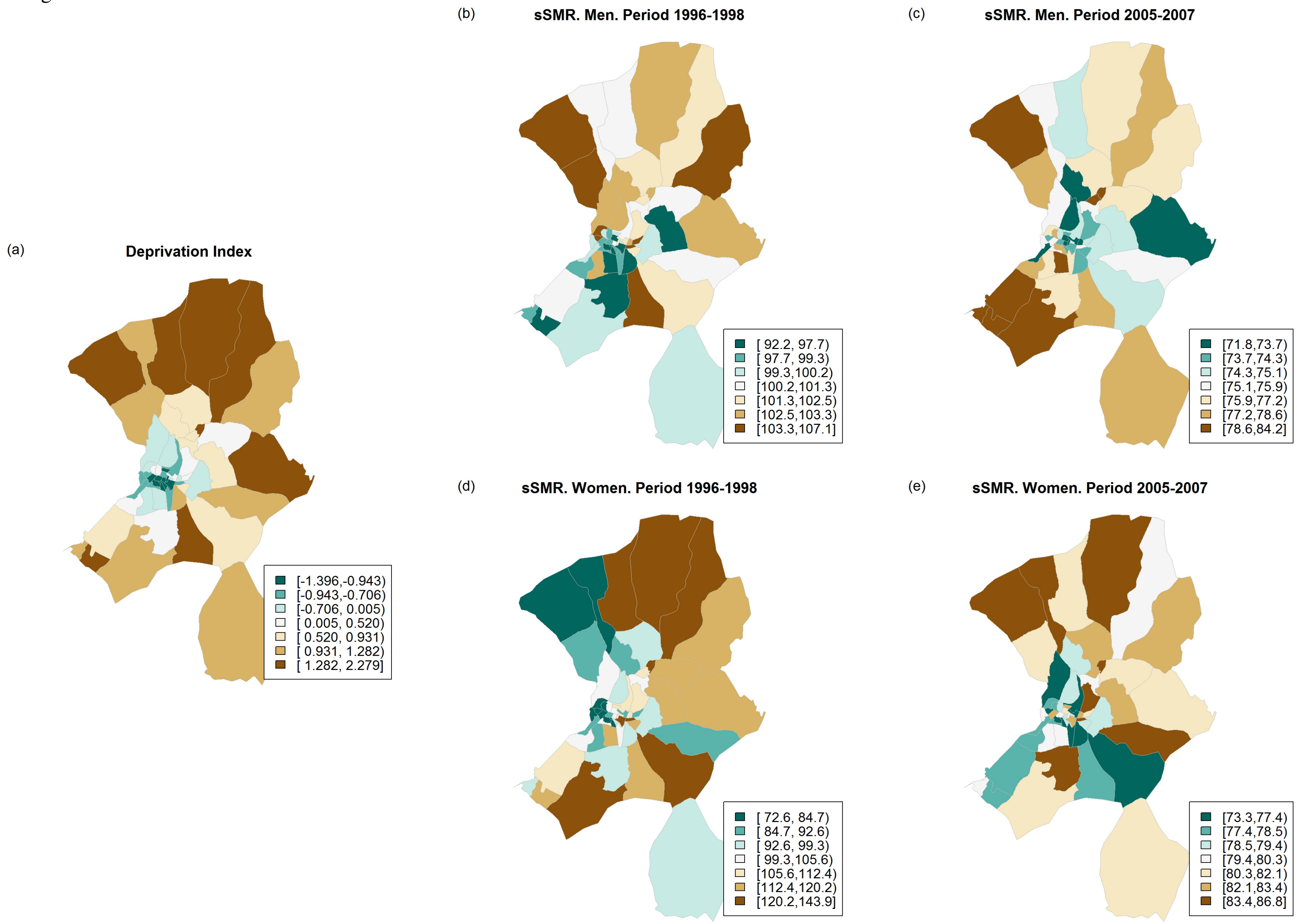

Figure 26. Distribution of deprivation index (a) and of the smoothed Standardised Mortality Ratios (sSMR) (b-e) for all-cause mortality, by period (1996-1998 and 2005-2007) and by sex in the city of San Sebastián. Green areas represent less socioeconomic deprivation and lower sSMR values. Brown areas represent greater socioeconomic deprivation and higher sSMR values.

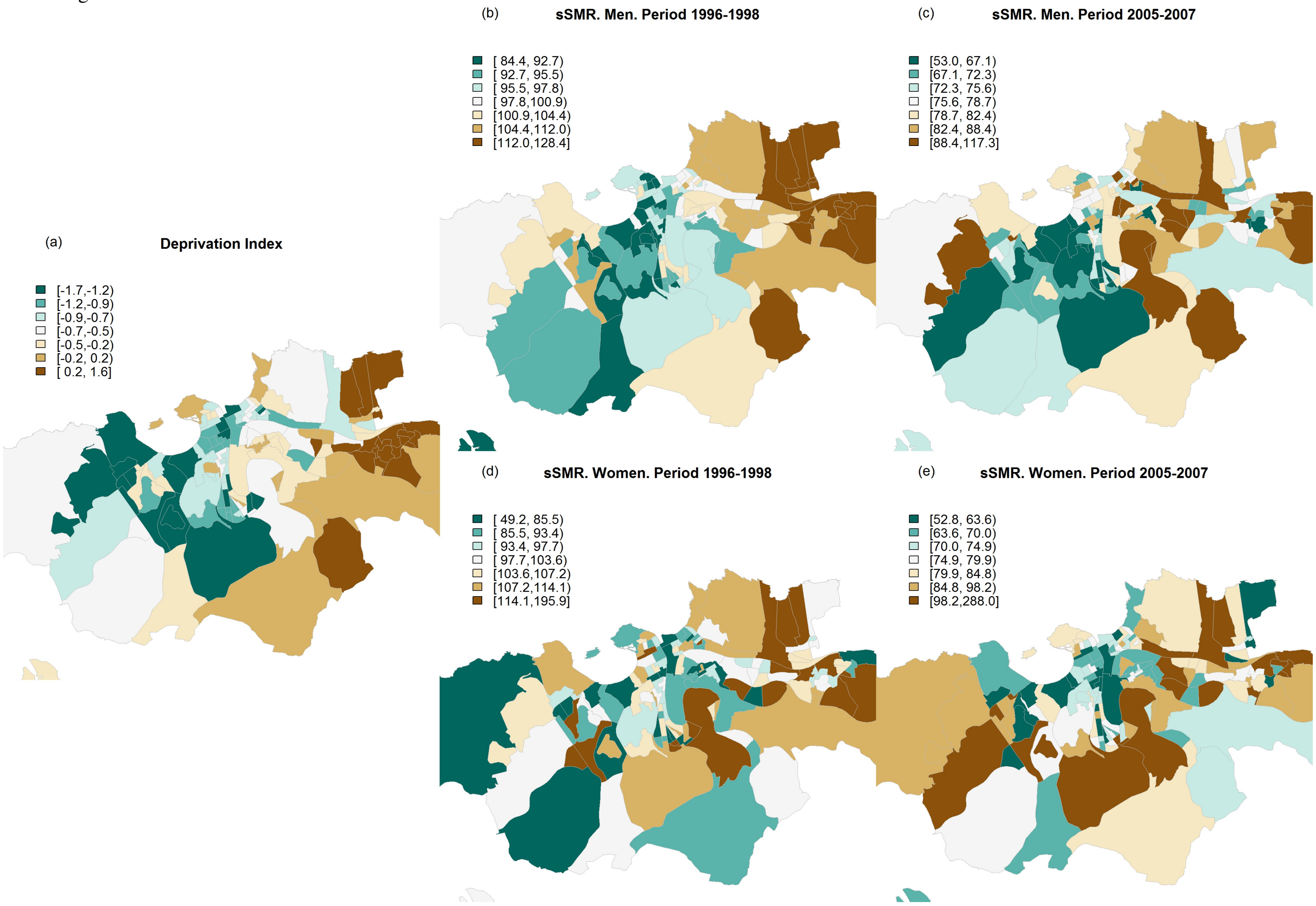

Figure 27. Distribution of deprivation index (a) and of the smoothed Standardised Mortality Ratios (sSMR) (b-e) for all-cause mortality, by period (1996-1998 and 2005-2007) and by sex in the city of Santa Cruz. Green areas represent less socioeconomic deprivation and lower sSMR values. Brown areas represent greater socioeconomic deprivation and higher sSMR values.

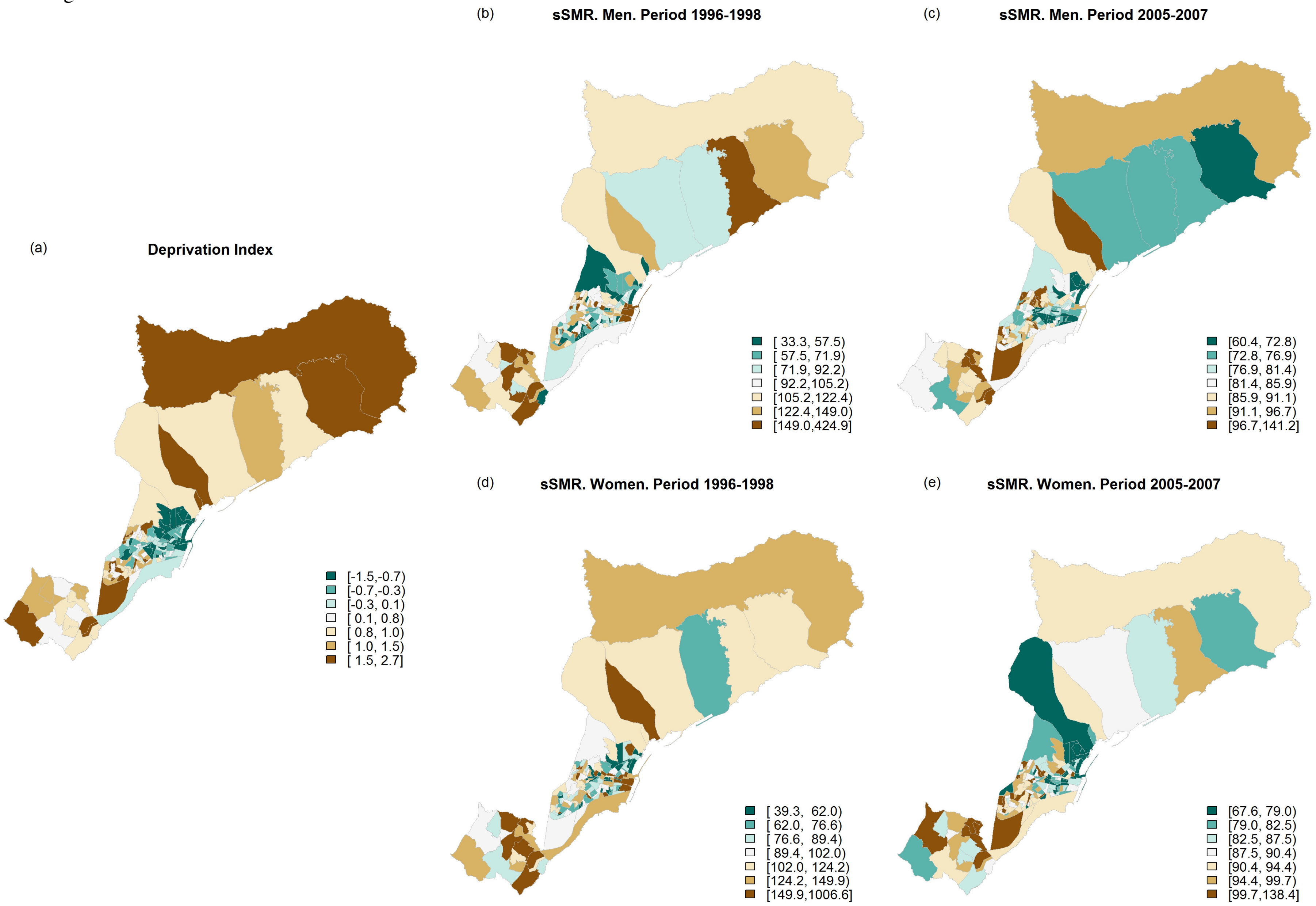

Figure 28. Distribution of deprivation index (a) and of the smoothed Standardised Mortality Ratios (sSMR) (b-e) for all-cause mortality, by period (1996-1998 and 2005-2007) and by sex in the city of Santiago. Green areas represent less socioeconomic deprivation and lower sSMR values. Brown areas represent greater socioeconomic deprivation and higher sSMR values.

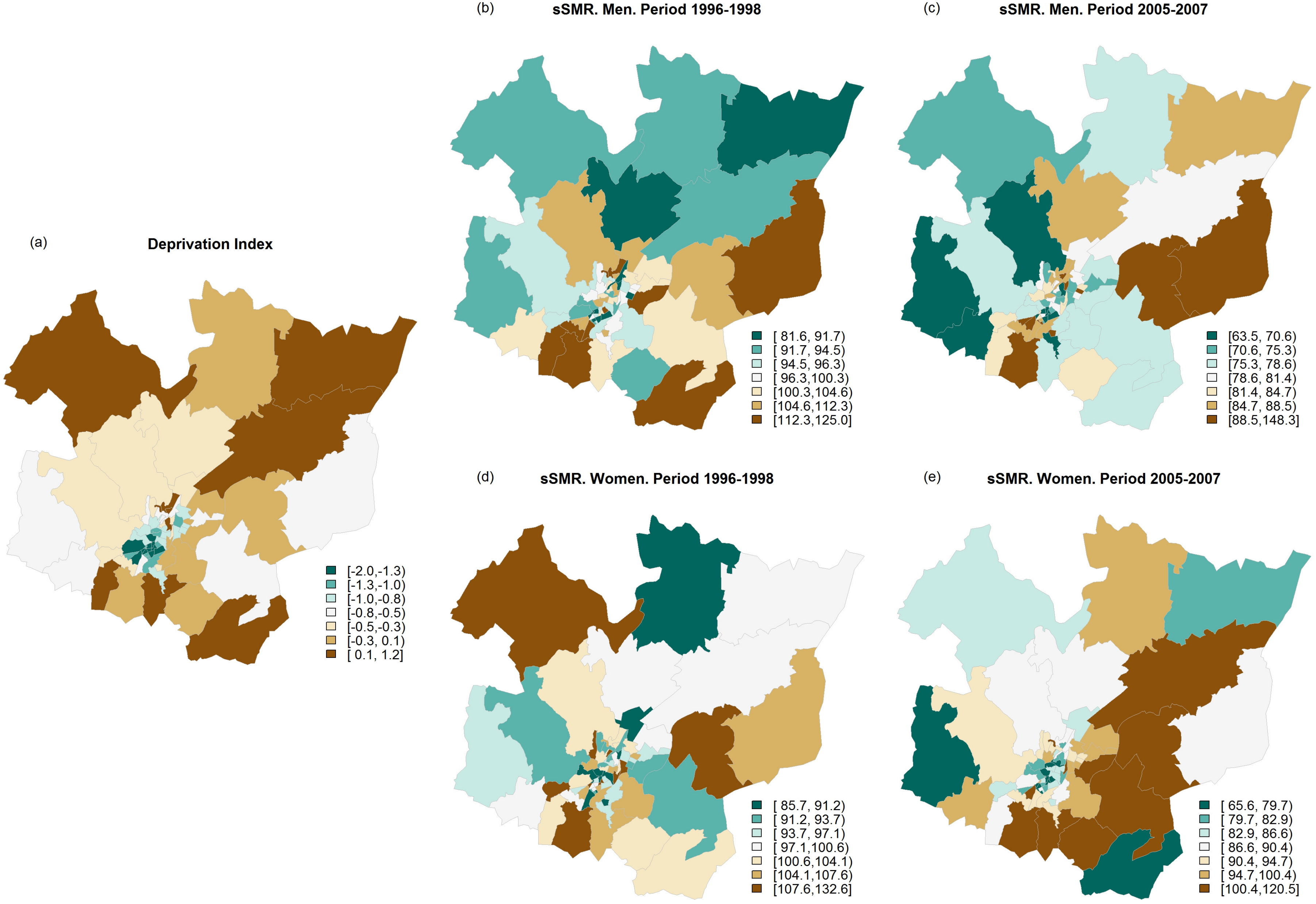

Figure 29. Distribution of deprivation index (a) and of the smoothed Standardised Mortality Ratios (sSMR) (b-e) for all-cause mortality, by period (1996-1998 and 2005-2007) and by sex in the city of Sevilla. Green areas represent less socioeconomic deprivation and lower sSMR values. Brown areas represent greater socioeconomic deprivation and higher sSMR values.

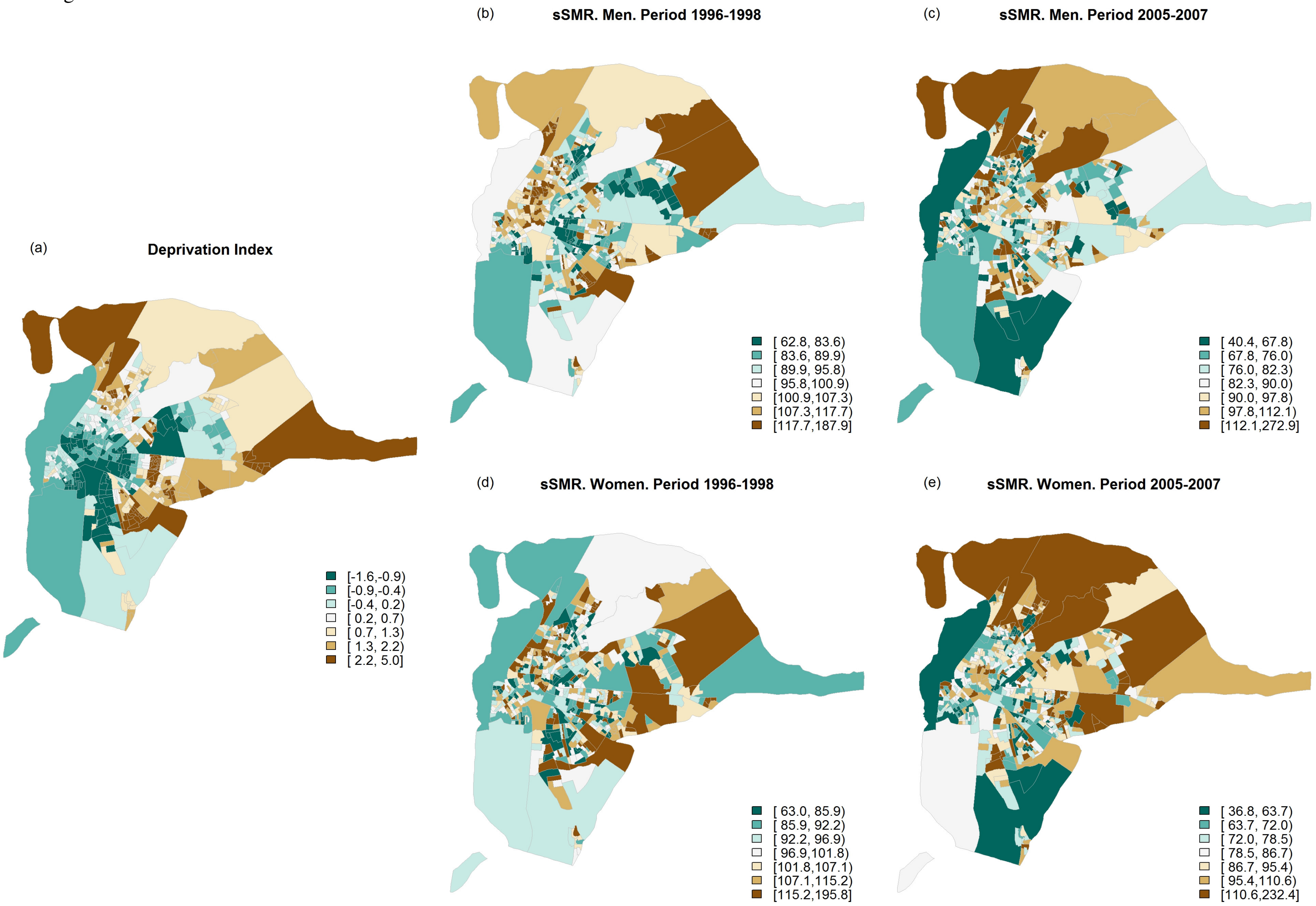

Figure 30. Distribution of deprivation index (a) and of the smoothed Standardised Mortality Ratios (sSMR) (b-e) for all-cause mortality, by period (1996-1998 and 2005-2007) and by sex in the city of Valencia. Green areas represent less socioeconomic deprivation and lower sSMR values. Brown areas represent greater socioeconomic deprivation and higher sSMR values.

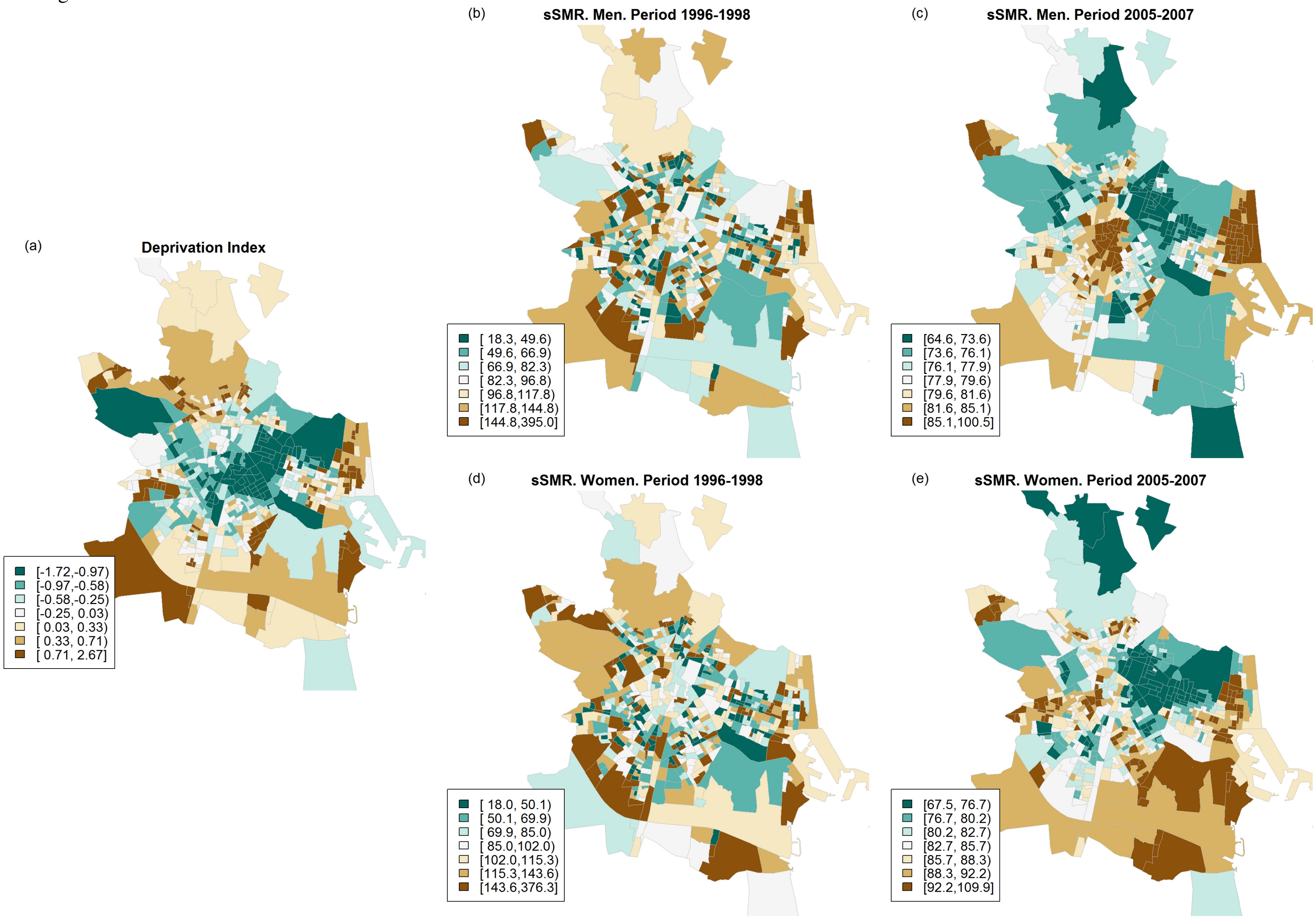

Figure 31. Distribution of deprivation index (a) and of the smoothed Standardised Mortality Ratios (sSMR) (b-e) for all-cause mortality, by period (1996-1998 and 2005-2007) and by sex in the city of Vigo. Green areas represent less socioeconomic deprivation and lower sSMR values. Brown areas represent greater socioeconomic deprivation and higher sSMR values.

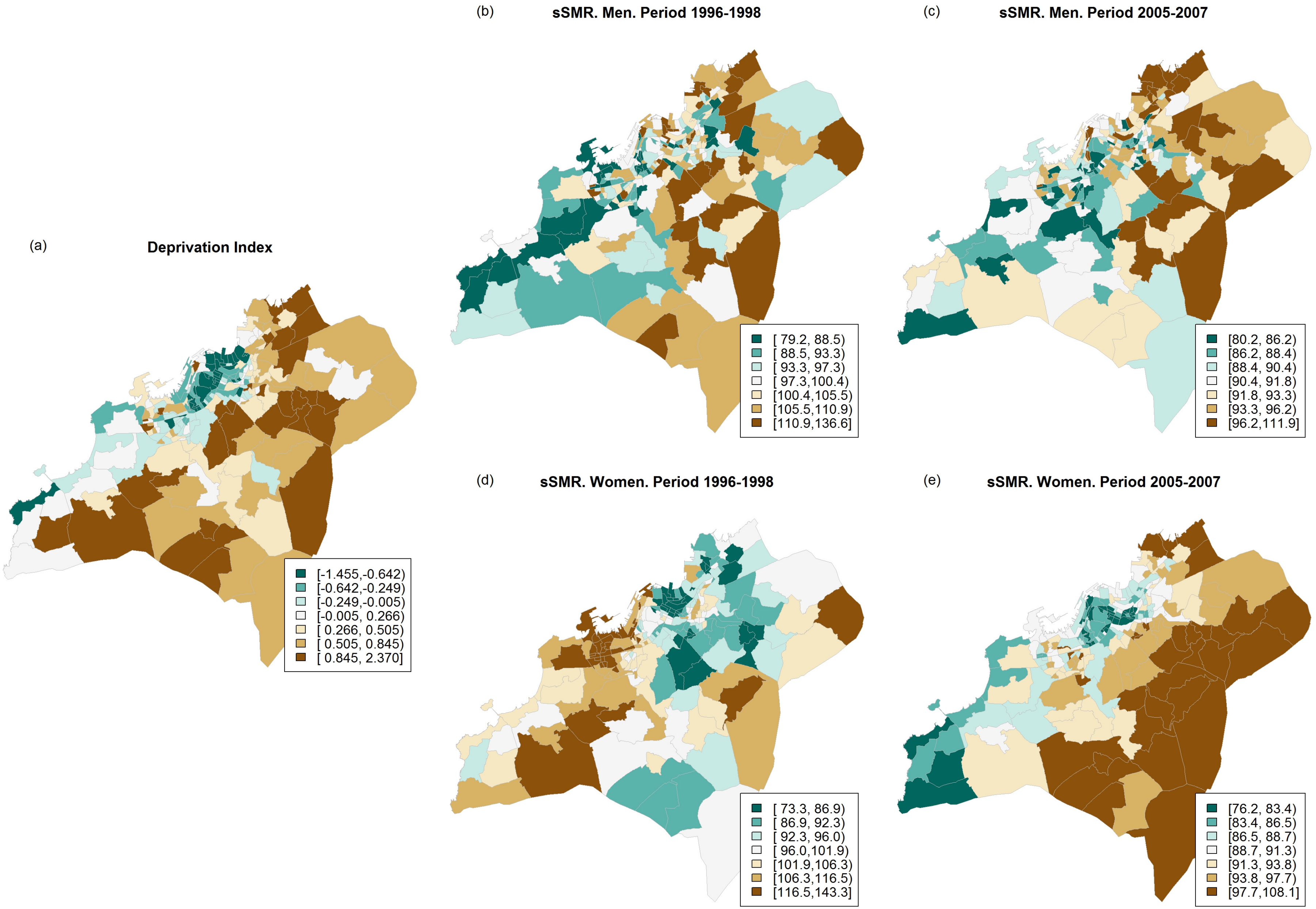

Figure 32. Distribution of deprivation index (a) and of the smoothed Standardised Mortality Ratios (sSMR) (b-e) for all-cause mortality, by period (1996-1998 and 2005-2007) and by sex in the city of Vitoria. Green areas represent less socioeconomic deprivation and lower sSMR values. Brown areas represent greater socioeconomic deprivation and higher sSMR values.

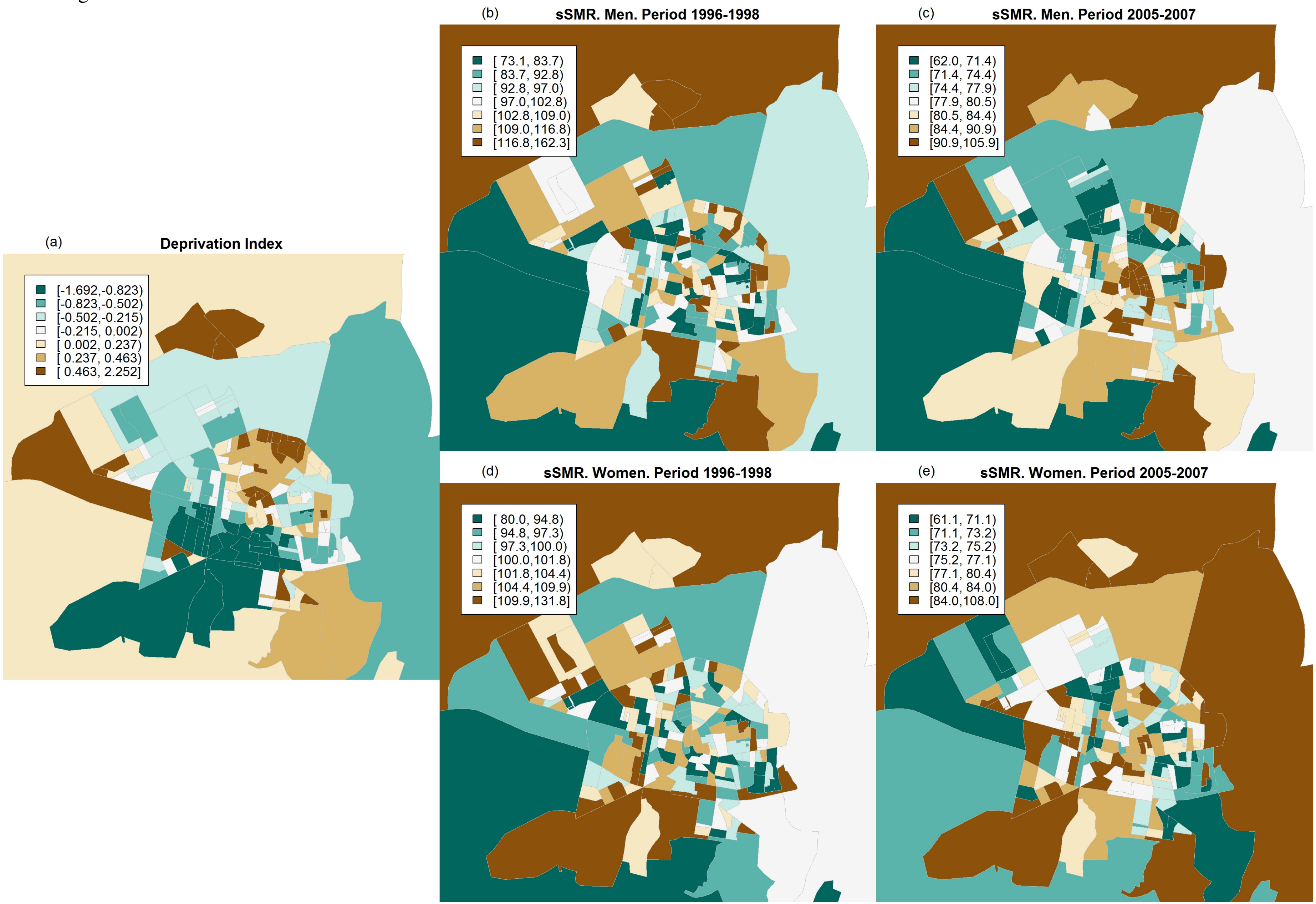

Figure 33. Distribution of deprivation index (a) and of the smoothed Standardised Mortality Ratios (sSMR) (b-e) for all-cause mortality, by period (1996-1998 and 2005-2007) and by sex in the city of Zaragoza. Green areas represent less socioeconomic deprivation and lower sSMR values. Brown areas represent greater socioeconomic deprivation and higher sSMR values.

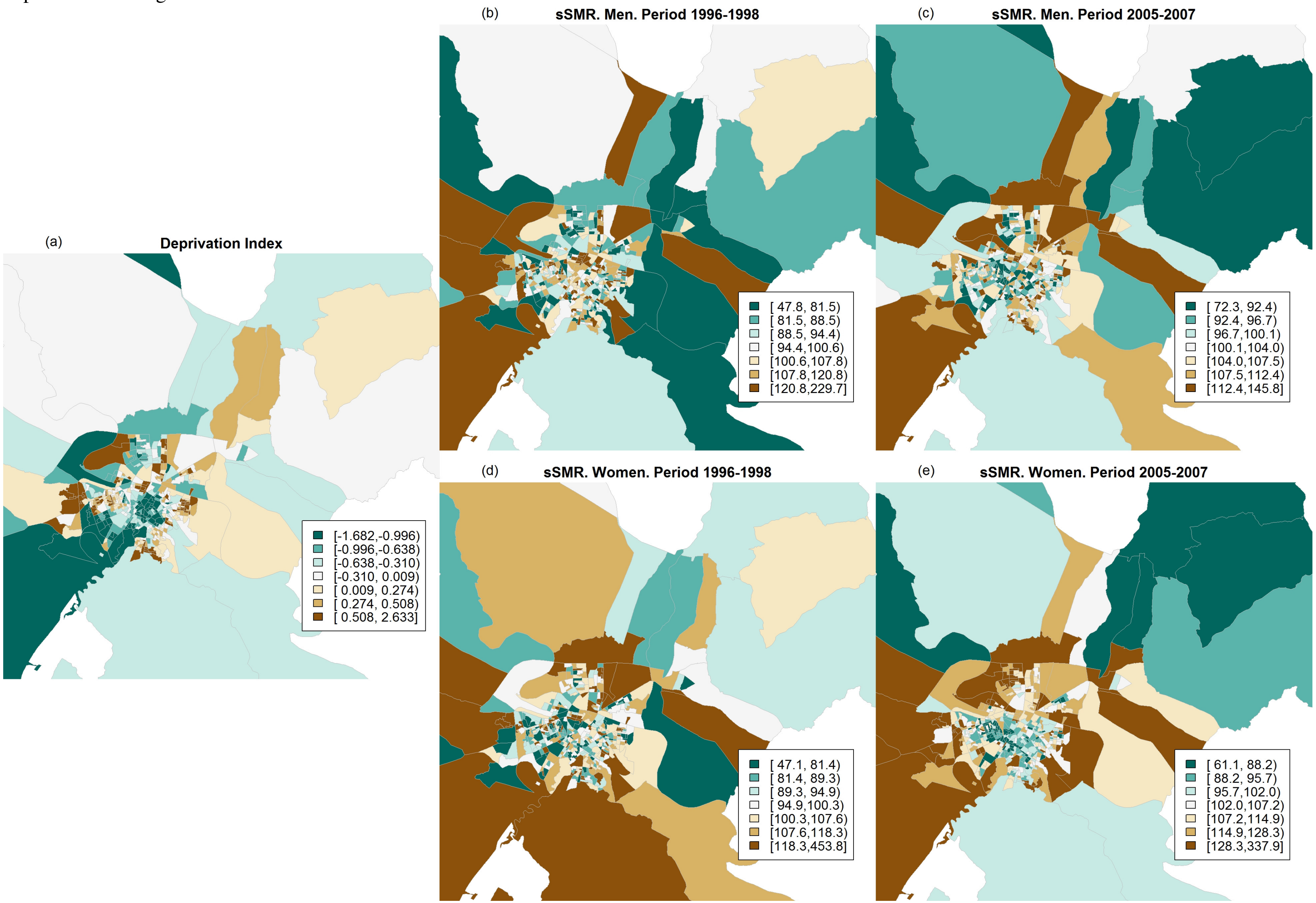

Supplement: Additional file 3: — Atlas of mortality in 33 Spanish cities (periods 1996–1998 and 2005–2007). The geographical distribution of the sSMR values in each of the cities and for each of the periods 1996–1998 (t = 1) and 2005–2006 (t = 2) has been represented in the form of maps of septiles. Together with these maps of mortality, we provide the geographical distribution of the deprivation index in each of the cities, again using septile maps. This makes it possible to check visually whether the spatial distribution of mortality has varied over time, and whether or not it is similar to the spatial distribution of deprivation. (PDF 42436 kb) [file 12889_2016_3190_MOESM3_ESM.pdf]
